# Potential molecular mechanism of exercise reversing insulin resistance and improving neurodegenerative diseases

**DOI:** 10.3389/fphys.2024.1337442

**Published:** 2024-05-16

**Authors:** Jiawen Shen, Xianping Wang, Minghui Wang, Hu Zhang

**Affiliations:** ^1^ Taizhou Hospital of Zhejiang Province Affiliated to Wenzhou Medical University, Taizhou, China; ^2^ School of Medicine, Taizhou University, Taizhou, China; ^3^ College of Sports Medicine, Wuhan Sports University, Wuhan, China

**Keywords:** exercise, insulin resistance, alzheimer’s disease, Parkinson’s disease, huntington’s disease, amyotrophic lateral sclerosis, multiple sclerosis

## Abstract

Neurodegenerative diseases are debilitating nervous system disorders attributed to various conditions such as body aging, gene mutations, genetic factors, and immune system disorders. Prominent neurodegenerative diseases include Alzheimer’s disease, Parkinson’s disease, Huntington’s disease, amyotrophic lateral sclerosis, and multiple sclerosis. Insulin resistance refers to the inability of the peripheral and central tissues of the body to respond to insulin and effectively regulate blood sugar levels. Insulin resistance has been observed in various neurodegenerative diseases and has been suggested to induce the occurrence, development, and exacerbation of neurodegenerative diseases. Furthermore, an increasing number of studies have suggested that reversing insulin resistance may be a critical intervention for the treatment of neurodegenerative diseases. Among the numerous measures available to improve insulin sensitivity, exercise is a widely accepted strategy due to its convenience, affordability, and significant impact on increasing insulin sensitivity. This review examines the association between neurodegenerative diseases and insulin resistance and highlights the molecular mechanisms by which exercise can reverse insulin resistance under these conditions. The focus was on regulating insulin resistance through exercise and providing practical ideas and suggestions for future research focused on exercise-induced insulin sensitivity in the context of neurodegenerative diseases.

## 1 Introduction

Research statistics suggest that the older population in certain countries will increase from approximately 11% in 1950 to an estimated 38% in 2030, and the number of older individuals will far exceed that of young individuals by 2050 (United Nations Population Division, 2002; Rudnicka et al., 2020). Chronic diseases may occur in many individuals. Moreover, the increasing incidence of neurodegenerative conditions has attracted the attention of researchers across various countries. The number of individuals with dementia, including preclinical Alzheimer’s disease (AD), preclinical AD, and AD, is estimated to collectively account for 22% of the global population aged ≥50 years (Gustavsson et al., 2023). Similarly, epidemiological statistics regarding neurodegenerative diseases indicate that the global proportion of patients with Parkinson’s disease (PD) may reach 12 million by 2040 (Dorsey et al., 2018).

**FIGURE 1:**
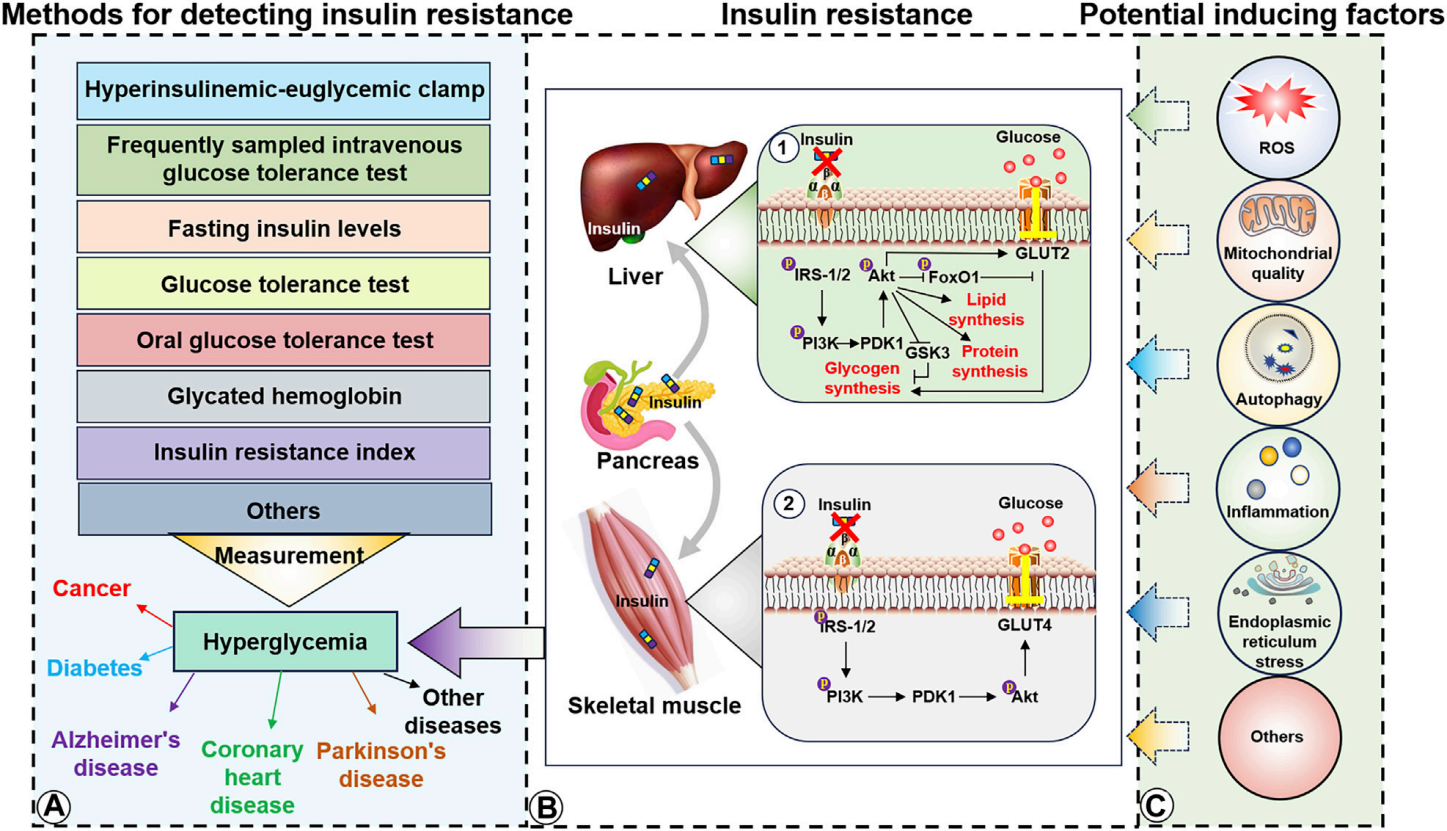
Detection methods, molecular mechanisms, and potential inducing factors of insulin resistance. **(A)**, Insulin detection methods and diseases potentially caused by hyperglycemia. **(B1)**, Insulin resistance in the liver induces IRS/PI3K/Akt to inhibit lipid, protein, and glycogen synthesis. **(B2)**, Insulin resistance in the skeletal muscle impairs the ability of GLUT4 to transport extra-cellular glucose into the muscle cells. **(C)**, Potential inducers of insulin resistance in the liver and skeletal muscle.

Currently, multiple sclerosis (MS) affects approximately 2.8 million individuals worldwide (The Lancet, 2021). In comparison, the global incidence rates of Huntington’s disease (HD) and amyotrophic lateral sclerosis (ALS) are estimated to be 0.48 and 4.42 cases per 100,000 individuals/year, respectively (Xu et al., 2020; Medina et al., 2022). In 2015, the worldwide cost of AD prevention and treatment alone reached US$957.56 billion, and this is expected to escalate to US$9.12 trillion by 2050 (Jia et al., 2018). Neurodegenerative diseases progressively deteriorate the quality of life of affected individuals and their families, and increase related medical costs and social burdens. Although monoclonal antibodies (Jia et al., 2018) and drugs such as levodopa (LeWitt, 2015), tetrabenazine (Frank et al., 2016), and edaravone (Rothstein, 2017) are regularly developed to treat common neurodegenerative diseases, few clinical trials and development cost issues associated with certain antibodies and drugs have limited the number of beneficiaries of these interventions. Therefore, identifying other safer and more reliable treatments and assisted rehabilitation pathways is vital to address these issues. Additionally, the occurrence, development, and progression of neurodegenerative diseases involve complex mechanisms. Therefore, the targeted exploration of relevant mechanisms and intervention targets may be critical for alleviating neurodegenerative diseases and improving patient recovery. Chronic inflammation caused by aging and chronic disease may lead to peripheral and central insulin resistance, ultimately affecting synaptic plasticity, neurotransmitter transport, and neuronal survival (Shonesy et al., 2012; Song et al., 2015). This increases the risk of neurological diseases and leads to alterations in learning, cognition, and dietary functions (Phillips et al., 2018; Wijtenburg et al., 2019; Cuperfain et al., 2020). Damage to early metabolic functions may be essential for the induction and exacerbation of neurological diseases (Fakih et al., 2022), among which insulin resistance is a standard feature (Reyes et al., 1984; Poewe and Seppi, 2017; Ruiz-Argüelles et al., 2018; Cordeiro et al., 2020; Kellar and Craft, 2020). Increasing insulin levels, enhancing insulin sensitivity, and regulating blood glucose levels can ameliorate neurodegenerative diseases (Wens et al., 2017; Aziz et al., 2020; Siddiqui et al., 2021) (Figure 1A). Therefore, a safe, effective, and economical insulin-sensitizing intervention may be a useful strategy for the prevention and treatment of neurodegenerative diseases. Presently, physical exercise is promoted in modern society as a method of healthcare and disease prevention and is extensively accepted by the public, it exhibits beneficial effects in the context of several chronic diseases. Furthermore, numerous studies have demonstrated that exercise can enhance insulin sensitivity (Angulo et al., 2020), thus suggesting that increasing insulin sensitivity through exercise may prevent and ameliorate neurodegenerative diseases. This review elucidates the relatively complex and critical molecular mechanisms involved in insulin resistance, neurodegenerative diseases, and the exercise-induced enhancement of insulin sensitivity. Here, we provide a scientific perspective regarding the significance of exercise-reversed insulin resistance in neurodegenerative diseases that will ultimately contribute to the prevention and treatment of neurodegenerative diseases in an increasingly older population.

## 2 Insulin resistance

After the discovery of insulin in 1921, a notable 1962 study by Rabinowitz and Zierler observed that exogenous insulin promotes glucose uptake in obese individuals. Furthermore, they observed that exogenous insulin enhanced the ability of adipose tissue to absorb glucose while simultaneously inhibiting the release of free fatty acids from adipose tissue, and these effects were significantly weaker in individuals with obesity than in the general population (Rabinowitz and Zierler, 1962). Following these prominent findings, insulin resistance gradually emerged as a popular research topic in the field of human health.

The advent of the Second Industrial Revolution rapidly increased the availability of goods and production for human life. This advancement has led to increased energy consumption and reduced physical labor that has resulted in obesity, type 2 diabetes-related metabolic diseases, and a high incidence of chronic diseases by providing adequate conditions for insulin resistance. Although the mechanisms underlying insulin resistance are still being explored, it can occur in multiple tissues and body organs, including the skeletal muscle, liver, brain, and fat tissue. Insulin is secreted by pancreatic islets and transported via the circulatory system to muscle tissue cells. Insulin molecules then target the insulin receptor (InsR), a cell-surface heterotetramer transmembrane receptor composed of two α- and two β-subunits. Subsequently, the intracellular PI3K/Akt/glucose transporter type 4 (GLUT4) signaling pathway is activated via recruitment and phosphorylation, and this facilitates the transport of plasma glucose into muscle and fat cells. Glucose transport provides energy for intracellular activities and helps to lower blood sugar levels (Figure 1B) (Lee and Pilch, 1994; Kahn, 1996; Pessin et al., 1999). Recent studies have reported that insulin resistance is primarily caused by the inability of insulin to effectively activate the insulin receptor substrate (IRS). This reduced IRS activation weakens GLUT4 function in the context of glucose transport. Additionally, the decreased ability of IRS-1/2 to effectively activate glycogen uptake in the liver hinders glycogen synthesis. Abnormalities ultimately lead to elevated blood glucose levels (Li et al., 2022). Although inflammation (Shoelson et al., 2006), oxidative stress (Evans et al., 2005), decreased mitochondrial function (Sangwung et al., 2020), circadian rhythm imbalance (Stenvers et al., 2019), intestinal flora disorder (Lee et al., 2020), and endoplasmic reticulum stress (Sims-Robinson et al., 2016) have been identified as essential insulin resistance factors, the specific causes of insulin resistance remain unclear (Figure 1C).

Furthermore, insulin resistance can alter the balance between glucose consumption and supply, ultimately resulting in higher blood sugar levels, and this in turn can promote diseases such as metabolic syndrome (Roberts et al., 2013), cancer (Roberts et al., 2013), coronary heart disease (Zhang Y. et al., 2022), AD (Talbot et al., 2012) and PD (Athauda and Foltynie, 2016). Currently, physical exercise, diet control, and drug interventions are the accepted primary means of increasing insulin sensitivity. Insulin resistance can be diagnosed using dynamic tests, simple indices, and biochemical markers (Borai et al., 2011), including dynamic hyperinsulinemic-euglycemic clamps, frequent sampling intravenous glucose tolerance tests, relatively simple fasting insulin levels and glucose tolerance tests, glycated hemoglobin, fasting blood glucose, insulin resistance index, and biological indicators such as leptin, adiponectin, triglycerides, high-density lipoprotein, and cholesterol, to make relevant predictions and judgments.

## 3 Exercise and nervous system insulin resistance

The human central nervous system requires approximately 20% (approximately 120–130 g) of the available glucose to maintain biological activities, including body movement and physiological reactions (Holliday, 1971). In the intricate dynamics of learning, memory formation, and many other critical brain functions, insulin is a vital facilitator that ensures the efficient uptake of glucose by the brain to adequately meet its heightened energy demands (Yano et al., 1991; Emmanuel et al., 2013). Previous studies incorrectly assumed that the brain is insensitive to insulin, as it does not produce insulin. However, later studies have established that the brain is regulated by insulin and demonstrated the occurrence of insulin resistance in the brain (Margolis and Altszuler, 1967; Havrankova et al., 1978). Furthermore, insulin intervention in the peripheral (Lv et al., 2020) and central nervous systems (Moosavi et al., 2006) of rats and mice can significantly alleviate neurological diseases, with insulin signaling activation as a potential molecular mechanism. Insulin has also been detected in the cerebrospinal fluid of humans and animals, thus further confirming its insulin’s role in the nervous system. Increases in peripheral insulin may be related to the selectivity, saturability, and active transport of the blood–brain barrier vascular endothelium (Margolis and Altszuler, 1967; Baura et al., 1993; Banks et al., 1997; Woods et al., 2003). Moreover, the expression of insulin mRNA in the brain tissue remains controversial (Arnold et al., 2018). Nevertheless, studies employing Cre/LoxP technology to knock out InsR in mouse brains have substantiated the critical role of insulin in the brain. These investigations demonstrated a significant increase in neuronal apoptosis and a significant decrease in downstream Akt and GSK3β phosphorylation levels, insulin resistance, and AD-related anxiety and depression (Brüning et al., 2000; Schubert et al., 2004; Kleinridders et al., 2015), thus indicating a potential link with high insulin levels in the synapses and its essential role (Werther et al., 1989; Abbott et al., 1999; Bockmann et al., 2002). Other researchers have observed InsR expression in different brain areas, including the cortex, hippocampus, hypothalamus, and cerebellum (Zhao et al., 2004; Dou et al., 2005; Fernandez and Torres-Alemán, 2012). Therefore, the current research suggests that insulin resistance occurs in the nervous system and may be associated with several neurological diseases.

Exercise can increase the energy consumption by the body to significantly accelerate glucose expenditure (via the liver, fat storage, and other tissues) and enhance glucose transport and utilization in cells that are crucial for brain cognition (Malin et al., 2022). The nervous system is susceptible to glucose, with insulin resistance resulting in poor learning and memory abilities and even neuronal degeneration. In line with this notion, a previous study indicated that insulin injections to elevate insulin levels enhanced learning and memory abilities in healthy young men and that exercise activities yielded similar outcomes (Zhang H. et al., 2022; Abbasi et al., 2023). Furthermore, short-term intermittent exercise in individuals with insulin resistance increases glucose uptake and metabolism in the brain. Additionally, an 8-week aerobic exercise intervention increased insulin sensitivity in individuals classified as overweight or sedentary obese with insulin resistance. Animal studies have revealed that middle-aged rats exhibit a certain degree of insulin resistance (Kuga et al., 2018). Moreover, exercise alleviates cognitive dysfunction in T2DM rats by activating the insulin signaling pathway (Rahmati et al., 2021). Finally, exercise-enhanced insulin signaling plays a vital role in neurodegenerative diseases (Mahalakshmi et al., 2020). According to these results, exercise effectively improves insulin resistance in the central nervous system and significantly positively affects learning, cognition, and prevention of neurological diseases.

## 4 Insulin resistance and neurodegenerative diseases

Neurodegenerative diseases are typically caused by aging. However, an increasing number of studies have observed that insulin signaling elicits abnormal changes involved in the occurrence and development of multiple neurodegenerative diseases. Moreover, the downregulation of InsR, failure of InsR to bind insulin, and impairment of the insulin cascade may occur in different neurodegenerative diseases. Therefore, we employed a disease-centered system to elucidate the mechanisms underlying IR in common neurodegenerative diseases.

### 4.1 Insulin resistance and Alzheimer’s disease (AD)

AD is a common neurodegenerative disease that significantly affects the quality of life of older adults worldwide. The present AD pathogenesis mechanism primarily involves excessive amyloid beta (Aβ) deposition and tau protein phosphorylation, and these factors are potentially related to insulin resistance (Kakoty et al., 2023a). Insulin resistance is significantly increased in the brains of normal-aging wild-type and APP/PS1 mice (Denver et al., 2018). Furthermore, a significant decrease in brain glucose utilization (assessed using 18F-FDG) has been reported in AD models (Waldron et al., 2015). Multiple studies have demonstrated that intranasal insulin intervention enhances cognitive performance in individuals with AD (Schiöth et al., 2012; Agrawal et al., 2018). Moreover, investigations conducted utilizing AD models further illuminate this issue, and although certain study outcomes may not be optimal, medical imaging findings have provided evidence of the association between insulin resistance and the degeneration of brain structures (Benedict et al., 2012; Cui et al., 2022).

Studies have demonstrated that insulin primarily influences neurons via the insulin/IRS/AKT pathway along with MAPK cascades. Insulin resistance may also induce and aggravate AD development by inhibiting the PI3K/Akt and Wnt signaling pathways and activating GSK3β, a crucial initiator of tau protein phosphorylation (Doble and Woodgett, 2003; Schubert et al., 2003). Additionally, abnormal tau gene expression is associated with impaired insulin signaling, whereas tau phosphorylation may be further enhanced by insulin resistance-induced increases in oxidative stress and inflammation (Schubert et al., 2004). Other researchers have observed that deletion and loss of function of tau protein can lead to metabolic/brain insulin resistance and damage the plasticity of hippocampal neurons (Marciniak et al., 2017). Moreover, insulin resistance can escalate tau protein hyperphosphorylation to a certain extent, thereby inducing and exacerbating AD and creating a vicious cycle. Furthermore, tau protein hyperphosphorylation is critical for promoting neurological diseases, whereas tau protein loss can result in the PD development of PD in mice (Lei et al., 2012). Therefore, reducing tau protein hyperphosphorylation by restoring insulin signaling may be a vital preventive measure against neurodegenerative diseases.

**FIGURE 2:**
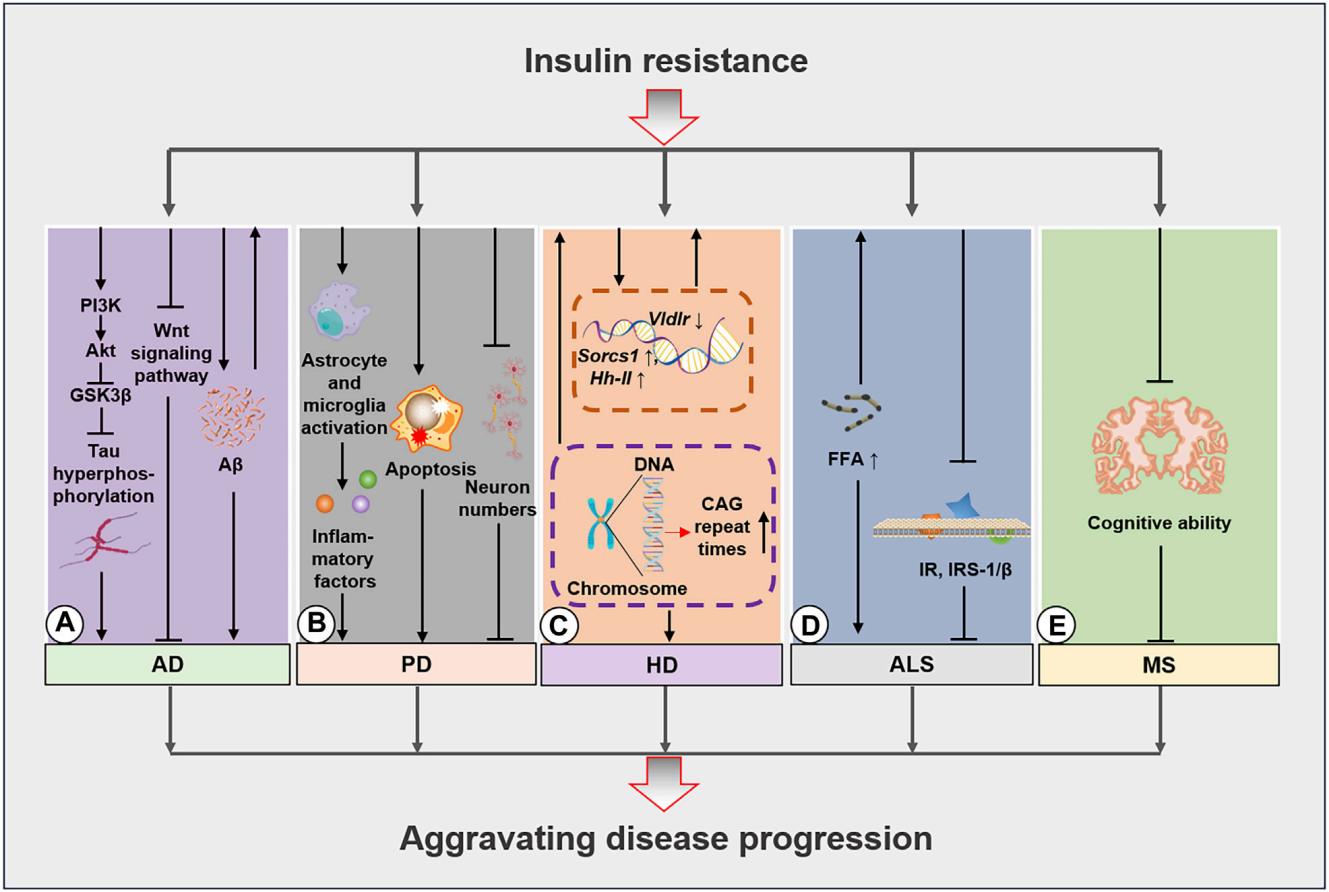
Molecular mechanisms of insulin resistance in neurodegenerative diseases. **(A)**, Insulin resistance aggravates AD development by inhibiting the PI3K/Akt/GSK3β pathway and inducing tau protein hyperphosphorylation. Additionally, insulin resistance impedes the Wnt signaling pathway and exacerbates Aβ deposition. These mechanisms further escalate the occurrence of insulin resistance. **(B)**, Insulin resistance activates starvation in glial cells and microglia induces apoptosis and lowers the proportion of neurons to worsen PD progression. **(C)**, HD caused by GCA sequence duplication leads to insulin resistance, while the regulation of Socrs1, Hh-II, and Vldlr in HD further amplifies disease progression. **(D)**, The increase in free fatty acid (FFA) levels in patients with ALS induces insulin resistance that in turn leads to decreases in IR and IRS-1/β expression. **(E)**, Insulin resistance further increases cognitive decline in patients with MS. AD, Alzheimer’s disease; PD, Parkinson’s disease; HD, Huntington’s disease; ALS, amyotrophic lateral sclerosis; Aβ, amyloid beta.

Aβ deposition in the human AD brain is positively correlated with peripheral insulin resistance, with insulin resistance being a preceding condition of Aβ deposition (Willette et al., 2015). Excessive Aβ inhibits the insulin binding ability of InsRs to some extent, and this further exacerbates insulin resistance (Xie et al., 2002) by activating JNK/TNF-α pathways and inducing IRS-1 phosphorylation (Bomfim et al., 2012; Yoon et al., 2012). These alterations, in turn, lead to increased Aβ deposition, thus forming a harmful loop. Intranasal insulin administration has decreased Aβ levels in 3xTg-AD mice brains (Chen et al., 2014). The reduction in Aβ accumulation may be due to the observation that activating the insulin signaling pathway improves Aβ precursor protein transport and metabolism via the Erk/MAPK signaling pathway and the regulation of α, β, and γ secretases (Gasparini et al., 2001; Gasparini et al., 2002; Cai et al., 2015). Conversely, many human studies have reported that the insulin-activated signaling pathways increase Aβ secretion levels in the cerebrospinal fluid (Watson et al., 2003) but accelerate Aβ clearance in the brain and reduce plasma Aβ levels (Figure 2A) (Reger et al., 2008a). Nevertheless, this contrasting result may be ascribed to the differences between the findings obtained in the models and tissue samples.

Further, the efficacy of insulin for efficacy in treating AD may be related to the *APOE-ε4* gene. Insulin intervention significantly improves in patients who do not carry the *APOE-ε4* gene, while those who exhibit significant improvement and general efficacy may even worsen (Reger et al., 2008b). Consequently, the development of insulin resistance treatments for AD may serve as a targeted treatment strategy. However, modulating insulin levels may require a comprehensive judgment, thus implying that non-insulin pathways to modulate the insulin signaling pathway may be a more helpful approach to alleviate AD.

### 4.2 Insulin resistance and Parkinson’s disease (PD)

PD is the second most common neurodegenerative disease, with a significantly increased occurrence among individuals aged >65 years. This trend may be related to the increased availability of industrial products and the changes in environmental factors. Patients with PD primarily present with resting tremors, body stiffness, abnormal walking posture, gait, and cognitive impairment. PD pathophysiology is likely linked to dysfunction and the loss of dopaminergic neurons, with α-synuclein accumulation, neuroinflammation, oxidative stress, mitochondrial dysfunction, and excessive consumption of neuromelanin as the possible triggers. However, in-depth research has suggested that insulin resistance may be a crucial factor in PD development and disease progression (Dai et al., 2023).

Studies of the brains of rat models of PD have revealed significantly decreased insulin levels and insulin resistance. Moreover, researchers have used antidiabetic drugs to improve the health status of patients with PD, thus highlighting the importance of alleviating the insulin signaling pathway in this disease (Bassil et al., 2022; Kakoty et al., 2023b). Researchers have applied mathematical modeling to elucidate insulin resistance in the brains of patients with PD, and the results have demonstrated that insulin resistance may develop gradually with disease severity and that insulin resistance markers are present in the striatum. Furthermore, the studies suggested that insulin resistance is significantly increased (Morris et al., 2011; Braatz and Coleman, 2015) by a high-fat diet, ultimately inducing behavioral changes and disease exacerbation in rats (Morris et al., 2010; Sharma and Taliyan, 2018). Insulin levels in peripheral plasma have also been observed to be significantly lower in patients with PD than those in healthy individuals (Sánchez-Gómez et al., 2020), and this possibly explains the prevalence of insulin resistance in the brain and blood circulation of patients with PD. In contrast, patients with new-onset PD do not exhibit any insulin resistance-related features (Aziz et al., 2020). Nevertheless, patients with diabetes are more likely to develop PD, potentially due to mitochondrial function and elevated oxidative stress levels caused by insulin resistance. Additionally, extensive loss of dopaminergic neurons may exacerbate memory impairment (Fiory et al., 2019). For example, PD-like changes have been observed in insulin-resistant PED/PEA-15 transgenic mouse models. In these mice, a 26% reduction in dopamine fibers and a 48% decline in dopamine levels were detected in the striatum (Perruolo et al., 2016), thus confirming the destructive relationship between PD and insulin resistance. Intranasal insulin intervention in a PD rat model was observed to improve cognitive ability via the insulin activation of the Akt/GSK3β signaling pathway (Figure 2B) (Yang et al., 2020). Other animal experiments have demonstrated that the insulin-stimulating peptide D-Ala2-GIP-glu-PAL increases insulin secretion and reduces insulin resistance. These changes led to several changes, including improving the slow movement and balance ability of mice, increasing the exploration ability of PD mice, decreasing astrocytes and microglia proportions in the substantia nigra, enhancing the number of neuronal synapses, lowering neuronal apoptosis, increasing the population of tyrosine hydroxylase-positive dopaminergic neurons in the substantia nigra, and increasing striatal tyrosine hydroxylase levels (Li et al., 2016). Conversely, a high-fat diet can severely escalate insulin resistance in mice with α-synuclein gene mutations (Rodriguez-Araujo et al., 2015). A series of studies have also indicated that a high-fat diet can induce and exacerbate dopaminergic degeneration (Bousquet et al., 2012), whereas individuals with metabolic syndrome were observed to exhibit a significantly increased PD risk (Park et al., 2021).

These findings imply that insulin resistance may be an important factor that triggers and complicates PD. Although insulin resistance in the PD brain is closely related to metabolic diseases, the underlying relationship between PD and insulin resistance remains unknown. Nonetheless, the amelioration of relevant metabolic diseases and insulin resistance may be essential for the prevention and management of PD.

### 4.3 Insulin resistance and Huntington’s disease (HD)

HD is a progressive neurodegenerative disease that primarily affects middle-aged and elderly adults. The major cause of HD is the unstable expansion of CAG repeats on the autosomal chromosome 4, which converts the disease-specific huntingtin protein (HTT) into mutated huntingtin protein (mHTT). Subsequent accumulation of this mutated protein in the brain amplifies the decline in neuronal function, ultimately leading to disease occurrence with a dominant genetic pattern (Bates et al., 2015). HD is also known as “Huntington’s chorea” due to the behavioral changes manifested by involuntary movement of the smaller trunk and face muscles, abnormal muscle tone, and hyperreflexia. Moreover, advancements in HD result in cognitive decline that is followed by difficulties in swallowing and speaking and ultimately death.

Studies in transgenic mice (R6/2 HD model) have determined that expanded CAG repeat sequences in the HD gene lead to significant reductions in glucagon and insulin levels in the pancreatic tissue of the α- and β-cells, respectively. Correspondingly, another study revealed that the decrease in glucagon and insulin levels among patients with diabetes was 10% and 15%, respectively, compared to levels in the control group. Furthermore, the number of CAG repeats is inversely associated with insulin sensitivity (Hurlbert et al., 1999; Björkqvist et al., 2006; Aziz et al., 2010). Research involving an HD transgenic mouse model has demonstrated impaired insulin expression and production in the mouse pancreas that is accompanied by reduced expression of key insulin gene transcription factors. Therefore, impaired pancreatic function may be a critical factor in the development of diabetes in this mouse model (Andreassen et al., 2002). Researchers have observed that restoring insulin signaling via SIRT2 inhibition provides neuronal protection (Luthi-Carter et al., 2010; Arora and Dey, 2016). Additionally, liquid chromatography-high-resolution mass spectrometry analysis revealed insulin resistance in the urine of R6/2 mice (Speziale et al., 2023). Transcriptomics investigations have also reported significant changes in several genes involved in glucose uptake and homeostasis (*Sorcs1*, *Hh-II*, and *Vldlr*), with a lower glucose uptake rate in affected tissues and subsequent development of insulin resistance (Chaves et al., 2017) (Figure 2C). Metformin administration has been reported to extend the lifespan of HD mouse models by 20% (Ma et al., 2007), thus signifying a relationship between insulin resistance and other diabetes-inducing factors in HD. Examination of normoglycemic patients with HD has also revealed abnormal blood glucose regulation, delayed insulin peak after oral glucose tolerance tests (OGTTs), and decreased insulin secretion levels (Lalić et al., 2008; Russo et al., 2013). In contrast to these findings, some studies have indicated that patients with HD do not exhibit an increased risk of diabetes and no significant differences in related metabolic indicators, histological manifestations, and insulin secretion levels were observed (Bacos et al., 2008; Boesgaard et al., 2009; Nambron et al., 2016).

Nevertheless, these studies primarily suggest that insulin resistance manifests during HD and that alleviating insulin resistance may mitigate HD progression. Moreover, HD progression may be related to the mHTT-induced inhibition of pancreatic cell function and reduction in the insulin sensitivity of InsRs in the body cells. Therefore, insulin resistance in patients with HD may be an outcome of HD progression, and the insulin resistance in turn worsens the disease course and patient health. Hence, improving insulin resistance may provide a feasible approach to reducing the adverse effects of HD progression.

### 4.4 Insulin resistance and amyotrophic lateral sclerosis (ALS)

ALS is a degenerative neurological disease that is mainly caused by the degradation and loss of central nervous system motor neurons controlling the skeletal muscles, eventually leading to skeletal muscle atrophy and death due to failure in swallowing and breathing.

Insulin resistance was detected in patients with ALS as early as 1984. However, whether defective InsR function or the emergence of receptor antagonists represents the specific underlying mechanism remains unclear (Reyes et al., 1984). Studies conducted during the same year demonstrated contrasting findings, wherein no signs of differential deterioration of glucose tolerance were detected in patients with ALS compared to healthy individuals as evidenced by their (125I)-insulin binding and OGTT results (Harno et al., 1984). Conversely, another study revealed that patients with ALS exhibited abnormal glucose tolerance and increased concentrations of free fatty acids which are important inducers of insulin resistance (Pradat et al., 2010). Subsequent studies confirmed that insulin resistance in patients with ALS may be associated with reduced InsR expression (Figure 2D) (Perurena and Festoff, 1987). A postmortem study revealed that InsR and IRS-1/β expressions were significantly reduced in the muscles and fibroblasts of patients with spinal cord and bulbar muscular atrophy, while insulin resistance was a critical indicator of disease severity (Nakatsuji et al., 2017). Furthermore, the presence of hypermetabolic diseases was determined to be a common characteristic among patients with ALS before disease onset and diagnosis (Tsai et al., 2019). Other researchers have suggested an alternative and potentially more reasonable explanation, where insulin resistance in patients with ALS may be closely linked to their limitations in performing daily activities due to their disease severity (Harris et al., 1986). Certain studies have also proposed that insulin resistance-induced metabolic differences can further exacerbate skeletal muscle atrophy during the disease course (de la Rubia Ortí et al., 2021). Finally, skeletal muscle atrophy and early impairment of insulin secretion may lead to hyperosmolar hyperglycemia in patients with late-stage ALS (Shimizu et al., 2011).

Considering these findings, insulin resistance may occur in ALS and may be an essential factor for inducing and exacerbating this disease as well as in predicting its incidence. Therefore, actively focusing on insulin resistance may prove valuable for the prevention and treatment of ALS.

### 4.5 Insulin resistance and multiple sclerosis (MS)

MS is a debilitating disorder of the central nervous system of unknown etiology. MS has been attributed to genetic and environmental factors and induces neuroinflammation, ultimately causing damage or degeneration in the brain and spinal cord. The major symptoms of MS include limb numbness, abnormalities in vision, cognition, speech, and even death.

In a metabolomic study of blood samples from 64 patients with MS, 33 patients exhibited insulin resistance based on their Quantose scores (Ruiz-Argüelles et al., 2018). Other studies have established that insulin resistance and metabolic syndrome are more common in patients with MS than they are in normal individuals (Soliman et al., 2020). In support of the previous finding, improving metabolic syndrome was found to play a positive role in preventing and treating MS (Haghikia and Gold, 2016). In contrast, OGTT results indicated that decreased insulin sensitivity due to postprandial hyperinsulinemia may not be directly related to a lack of exercise and higher inflammation levels (Penesova et al., 2015). Nevertheless, studies have demonstrated that patients with MS, with or without diabetes, present with insulin resistance and a significant reduction in insulin secretion (Katsuki et al., 1998; Kouchaki et al., 2017). Researchers have also demonstrated that insulin resistance is associated with cognitive decline in patients with MS, with a cognitive decline rate of 67.56% in patients with concurrent MS and insulin resistance (Khalili et al., 2020; Ayromlou et al., 2023). Furthermore, the onset of IR may precede blood lipid changes in the early stages of MS (Radikova et al., 2018). Additionally, 12 weeks of high-intensity aerobic and resistance training improved glucose tolerance in patients with MS, while 10 weeks of aerobic exercise training reduced insulin resistance in women with MS (Sadegh and Golestany, 2017; Wens et al., 2017). Conversely, certain studies have demonstrated that glucose control in patients with MS was not enhanced after 24 weeks of combined endurance and resistance training (Wens et al., 2015). Although the prevalence of type II diabetes mellitus (T2DM) in the MS population is <10%, it rate is still higher than that in the general population (Giannopapas et al., 2023). Moreover, patients with T2DM in the MS population display significantly reduced levels of insulin secretion compared to that of patients without T2DM (Katsuki et al., 1998). As observed in some previously mentioned neurodegenerative diseases, intranasal insulin intervention significantly augmented the cognitive abilities of patients with MS, thus providing further supportive evidence of mechanism of the insulin resistance in patients with MS (Mowry et al., 2017). Based on these results, insulin resistance may exist in MS, and ameliorating it may attenuate MS progression and serve as a useful strategy for the prevention and management of MS (Figure 2E).

## 5 Exercise reduces insulin resistance and improves neurological diseases

Limited studies have shown that exercise can effectively reduce central and peripheral insulin resistance to improve neurodegenerative diseases. However, most of these studies have focused on AD, PD, and MS disease models and populations.

Relevant research has found that 6 weeks of swimming and resistance exercise in D-galactose-induced AD-like models can increase the circulating insulin-like growth factor 1 (IGF-1) levels in the body, leading to improvements in cognitive abilities (Özbeyli et al., 2017). Moreover, in an AD rat model, induced by exogenous Streptozotocin (STZ) and Aβ injection, treadmill exercise significantly activated the insulin signaling pathway in the hippocampus, reduced peripheral blood glucose levels, and improved cognitive ability (Kang and Cho, 2014; Farokhi Larijani et al., 2023). Notably, these changes also appear in transgenic AD mice (Um et al., 2008; Kim et al., 2022). However, in a 16-week study on patients with AD, aerobic exercise resulted in a decrease in blood glucose levels but did not improve insulin resistance. In another 24-week intervention in patients with AD, a decrease in insulin resistance was observed. The reason for this difference in results may be related to the difference in intervention duration and sample size between the two studies. Previous studies have shown that exercise can partially suppress insulin resistance in both AD models and humans. However, human studies may require larger sample sizes and more in-depth investigations.

Studies have shown that aerobic exercise alone and lower-intensity balance exercises are not effective in reducing insulin resistance and IGF-1 levels in the PD population (Szymura et al., 2020; Soke et al., 2021). However, combining aerobic and strength exercises along with multi-modal exercise has been demonstrated to significantly reduce fasting blood glucose levels and increase IGF-1 levels and cognitive abilities (Krumpolec et al., 2017; Stuckenschneider et al., 2021). The mixed exercise model thus appears to be more effective in lowering blood sugar levels and improving peripheral insulin resistance in individuals with PD.

In MS models, swimming and free-wheel exercise can activate the IGF-1/Akt/FOXO3a and IGF-1 and PI3K/AKT signalling pathways in the skeletal muscle and spinal cord tissue, respectively (Kaspar et al., 2005; Cieminski et al., 2021). Exercise in patients with MS has shown good anti-insulin resistance effects. Among these, home exercise, swimming, aerobic exercise, and resistance exercise, as well as a variety of combined exercise training programs, can improve glucose tolerance and reduce blood sugar levels and insulin resistance. Varying degrees of improvement have also been observed (Patyn, 2014; Wens et al., 2015; Sadegh and Golestany, 2017; Wens et al., 2017; Ispoglou et al., 2023).

Exercise plays a positive role in neurodegenerative diseases by improving peripheral and central insulin resistance (Table 1). However, research on other neurodegenerative diseases is lacking.

**TABLE 1:** Exercise improves insulin resistance in neurodegenerative diseases.

Model	Exercise model	Exercise dose	Changes in indicators related to insulin resistance	Source and tests	References
AD (patients)	Aerobic exercise	60 min/time, 3 times/week, 16 weeks	HDL↑; Blood glucose and insulin→	Plasma	Jensen et al. (2020)
AD (patients)	Aerobic exercise	45–60 min/time, 4 times/week, 24 weeks	Insulin sensitivity↑	Plasma	Baker et al. (2010)
AD (3xTg AD mice)	Treadmill running	13 m/min, 50 min/time, 6 times/week, 12 weeks	Insulin signaling↑	Hippocampus	Kim et al. (2022)
AD (Aβ-induced AD rats)	Treadmill running	60 min/time, 7 times/week, 4 weeks	Glucose level↓; Cognitive function↑	Plasma	Farokhi Larijani et al. (2023)
AD (STZ-induced AD model)	Treadmill running	14 m/min, 30 min/time, 5 times/week, 6 weeks	Insulin signaling pathway and cognitive function↑	Hippocampus	Kang and Cho (2014)
AD model (NSE/APPsw-Tg mice)	Treadmill running	22 m/min, 60 min/time, 5 times/week, 16 weeks	Blood glucose↓; GLUT1 and cognitive function↑	Plasma	Um et al. (2008)
AD-like model (D-galactose induced Wistar rat)	Swimming and resistance exercise	60 min/time, 3 times/week, 6 weeks	IGF-I and cognitive function↑	Plasma and brain	Özbeyli et al. (2017)
PD patients	Aerobic exercise	30 min/time, 3 times/week, 8 weeks	IGF-1→	Plasma	Soke et al. (2021)
PD patients	Aerobic-strength training	60 min/time, 3 times/week, 12 weeks	Fasting and 2 h blood glucose↓	Plasma	Krumpolec et al. (2017)
PD patients	Balance training	60 min/time, 3 times/week, 12 weeks	IGF-1→	Plasma	Szymura et al. (2020)
PD patients	Multimodal exercise intervention methods	60 min/time, 2 times/week, 8 weeks	IGF-1↑; Cognitive function→	Plasma and behavior	Stuckenschneider et al. (2021)
MS patients	Home exercise	2 time/week, 24 weeks	IGF-1↑	Plasma	Ispoglou et al. (2023)
MS patients	Aerobic exercise and resistance exercise	5 time/2 weeks 24 weeks	Fasting blood glucose, tAUC, and OGTT↓; GT and GLUT4↑	Plasma	Wens et al. (2017)
MS patients	Joint training plan	5 time/2 weeks, 24 weeks	Fasting blood glucose, insulin, and tAUC→	Plasma	Wens et al. (2015)
MS patients	Swimming	10 weeks	Fasting glucose, and insulin resistance↓	Plasma	Sadegh and Golestany (2017)
MS patients	Resistance and high-intensity interval training	12 weeks	glucose tolerance↑	Plasma	Patyn (2014)
MS model (SOD1 transgenic mice)	Swimming	30 min/time, 5 times/week, 6.5 weeks	IGF-1/Akt/FOXO3a↑	Muscle	Cieminski et al. (2021)
MS model (SOD1 transgenic mice)	Voluntary wheel running	6 or 12 h	IGF-1 and PI3K/AKT↑	Spinal cord	Kaspar et al. (2005)

tAUC, total area under the glucose curve; GT, glucose tolerance; IGF-1, insulin-like growth factor 1; GLUT, 1/4, glucose transporter type 1/4; ↑, represents upregulation; ↓, represents downregulation; →, represents no change.

## 6 Potential mechanisms of exercise in improving insulin resistance

Exercise has long been recognized as an important strategy for preventing and treating chronic diseases and accelerating recovery. However, the molecular mechanisms underlying these beneficial effects of exercise require further systematic exploration, as this involves a broad concept of motion. Considering that insulin resistance has been established to occur in neurodegenerative diseases, exercise may represent a feasible intervention to improve insulin resistance, and its molecular mechanisms potentially involve mitochondrial quality, inflammation, autophagy, endoplasmic reticulum stress, and reactive oxygen species. Therefore, we summarized the triggering factors of exercise reversing insulin resistance and provided possible mechanisms by which exercise reverses insulin resistance in neurodegenerative diseases.

### 6.1 Mitochondria quality

**FIGURE 3:**
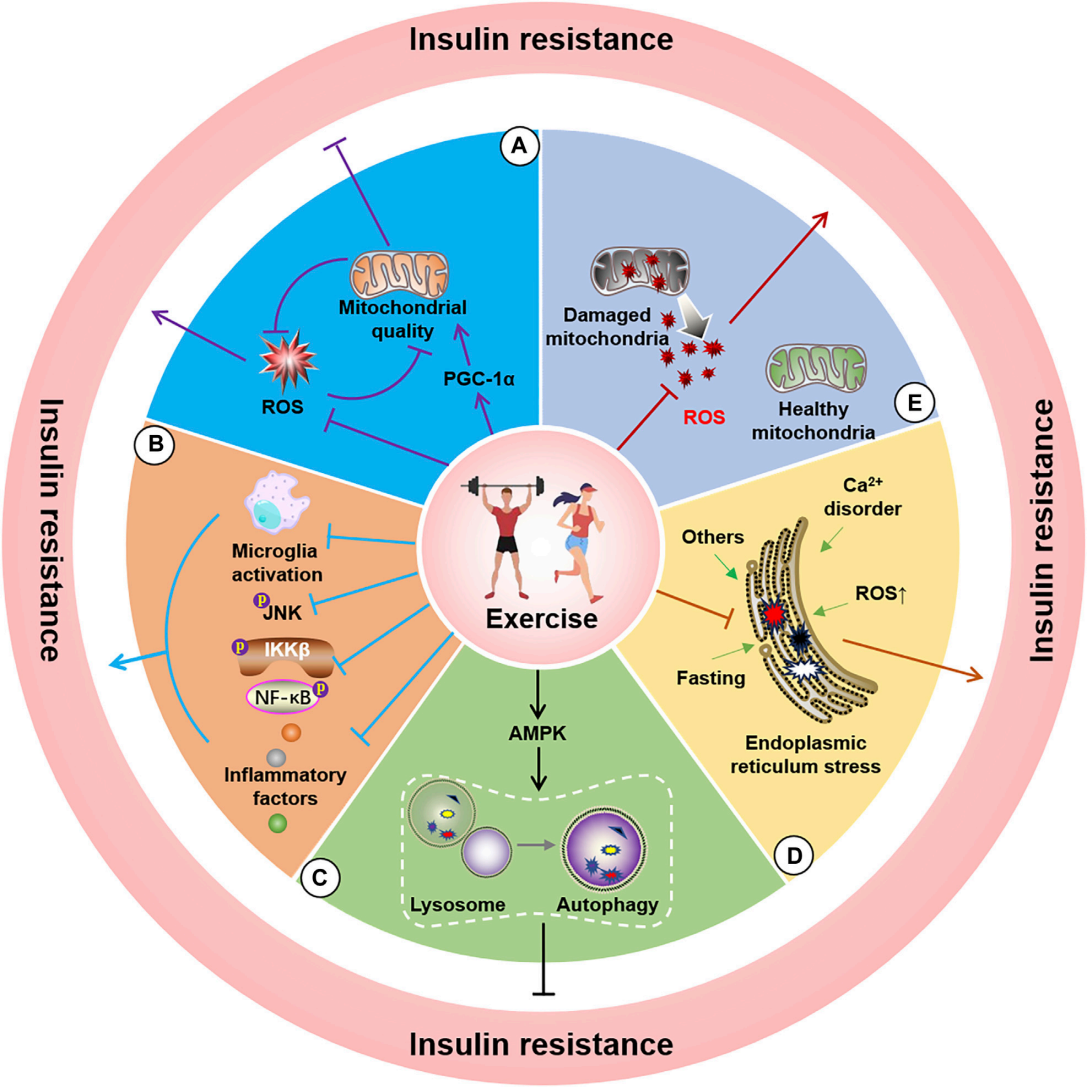
Potential molecular mechanisms of exercise for improving insulin resistance. A, Exercise alleviates insulin resistance by improving mitochondrial quality **(A)**, reducing ROS **(B)**, decreasing endoplasmic reticulum stress **(C)**, activating AMPK to induce autophagy **(D)**, and inhibiting microglial activation and JNK and NF-κB phosphorylation as well as lowering neuroinflammatory factors **(E)**.

Mitochondria are pivotal organelles involved in cellular energy metabolism and are more abundant in tissues with strong energy demands. Mitochondria primarily generate ATP via the oxidative phosphorylation pathway for regular physiological functions. The insulin/IRS/GLUT4 pathway is the main route by which cells take up glucose and convert energy through mitochondrial processing. Although mitochondrial oxidative phosphorylation theoretically follows insulin-mediated glucose uptake, a high-glucose-induced insulin model in primary cortical neurons indicated that mitochondrial dysfunction may be associated with insulin resistance (Peng et al., 2016), ultimately leading to acute insulin resistance in mice (Lee et al., 2021). For example, knockout of the mitophagy-related protein FNDC1 increases susceptibility to obesity-induced insulin resistance (Wu et al., 2019). Other studies have demonstrated that mitochondrial dysfunction may be a crucial component in the development of IR (Hoeks and Schrauwen, 2012). Furthermore, skeletal muscle insulin resistance was significantly reduced after transient transfection with the mitochondrial quality regulatory protein PGC-1α, a known regulator of mitochondrial quantity and respiration (Benton et al., 2008; Pagel-Langenickel et al., 2008). In contrast, skeletal muscle studies have indicated that insulin resistance may not be a consequence of decreased mitochondrial numbers, although the mitochondrial function is vital for the action of insulin, insulin resistance does not arise when the mitochondrial electron transport chain is disrupted (Han et al., 2011; Holloszy, 2013). Insulin resistance leads to deterioration of mitochondrial function and quality. This alteration can be improved by insulin treatment, with the mitochondrial proteome as a potential mechanism (Zabielski et al., 2016). Moreover, mitochondrial dysfunction in the presence of insulin resistance elevates levels of reactive oxygen species (ROS), thus creating a vicious cycle that further aggravates insulin resistance (Figure 3A) (Ruegsegger et al., 2018; Ayer et al., 2022). Physical activity is considered the dominant player in the link between insulin resistance and mitochondrial respiration, thus implying that regulating and monitoring the aerobic capacity may be critical for managing mitochondrial respiration-induced insulin resistance (Dela and Helge, 2013). Although the relationship between mitochondria and insulin is complex, in-depth research examining the relevant molecular mechanisms may be beneficial to reduce insulin resistance by regulating mitochondria. Conversely, some studies have demonstrated that improving mitochondrial proportion and respiratory function through exercise may not alleviate insulin resistance in individuals with obesity (Hey-Mogensen et al., 2010).

Although specific differences exist in glucose uptake between the nervous system and peripheral tissues, exercise may ameliorate mitochondrial damage (Park et al., 2018), enhance mitochondrial function (Ruegsegger et al., 2019), reduce ROS levels (Ayer et al., 2022), and regulate mRNA translation. The comprehensive beneficial effects of exercise (Jannig and Ruas, 2017) suggest its potential as a crucial approach for improving insulin resistance by targeting mitochondrial quality and ultimately mitigating neurodegenerative diseases.

### 6.2 Inflammation

Inflammation induces and aggravates numerous diseases, and several studies have highlighted it as an important promoter of insulin resistance. Inflammation-induced insulin resistance in the nervous system predominantly occurs due to increased levels of inflammatory factors such as ROS, endoplasmic reticulum stress, TNF-α/TNFR1, PKCs, and LPS/TLRs activation of intracellular JNK and IKKβ/NF-κB. These inflammatory cascades further inhibit IRS-1, ultimately leading to insulin resistance. Among these inflammatory pathways, inflammation induced by microglial activation has been established as a critical component in inducing insulin resistance (Doust et al., 2022), and studies have demonstrated that inhibiting microglial activation can restore insulin resistance-related cognitive decline (Chunchai et al., 2018). Moreover, the presence or absence of the components of the inflammatory cascade can improve insulin resistance. For example, LPS-induced inflammation is a key tool in animal and cellular modelling of neuroinflammation. LPS has been suggested to cause insulin resistance in the central nervous system by affecting JNK activation (Rorato et al., 2017). Furthermore, TLR4 knockout was observed to reduce high-fat diet-induced insulin resistance in mice (Shi et al., 2006), and inhibiting central IKKβ/NF-κB decreased insulin resistance in the brain (Figure 3B) (Benzler et al., 2015). Moreover, studies have demonstrated that peripheral inflammation-induced central insulin resistance can be mitigated by inhibiting inflammation in the central nervous system (Milanski et al., 2012). Research involving inflammation-mediated insulin resistance has also shown a close link between peripheral diseases and the nervous system. Several studies have revealed that obesity, a prominent chronic inflammation promoter in the body, is associated with a significantly increased risk of developing neurodegenerative diseases (Spielman et al., 2014). Reasonable physical exercise can provide a valuable means of inhibiting inflammation and microglial activation (Wang et al., 2016), with confirmatory indicating that exercise can prevent and treat neurological diseases by inhibiting inflammation-mediated insulin resistance. For example, studies have established that swimming can lower insulin resistance in the mouse hippocampus by decreasing nervous system inflammation (Zhang H. et al., 2022), whereas resistance exercise training has been reported to effectively alleviate neuroinflammation in the mouse brain (Kelty et al., 2019). Furthermore, exercise-induced suppression of inflammation has yielded positive effects in the context of neurodegenerative diseases, such as AD and PD, and these benefits are closely related to the mitigation of insulin resistance (Li et al., 2022; Wang et al., 2023). Therefore, inflammation-regulated insulin resistance may be associated with mechanisms acting against neurodegenerative changes, thus indicating that exercise interventions targeting the reduction of inflammation levels may represent a useful strategy.

### 6.3 Autophagy

Autophagy is a classical mechanism for the self-degradation of organelles and misfolded proteins damaged by aging. Abnormal autophagy levels have been reported in various diseases, thus indicating that regulation of autophagy is a potential mechanism for the prevention and treatment of numerous diseases. Autophagy encompasses several autophagy-related proteins that form circular vesicles in a specific sequence to engulf components that require degradation, and then combine with lysosomes, ultimately leading to the breakdown of the components into essential substances that are made available for recycling. However, the excessive activation or inhibition of autophagy may trigger certain diseases. For example, certain studies have linked knockout of the autophagy gene ATG16L1 to insulin resistance (Frendo-Cumbo et al., 2019). Another study found that reducing miR-188, an inhibitor of the target autophagy-related gene Atg12, enhanced insulin resistance (Liu et al., 2020). Additionally, impeding the activation of mitophagy and the Sirt1/AMPK-PGC1α signaling pathway by using miR-302 in human podocytes has significantly reduced insulin resistance in HD (Chang et al., 2021). Cell experiments in human podocytes also demonstrated that autophagy activation can significantly alleviate insulin resistance attributed to high glucose levels (Xin et al., 2016). In contrast, minimizing autophagy and inducing autophagic flux can lead to insulin resistance in the body (Cai et al., 2018; Oh et al., 2023). Researchers have also demonstrated that the regulation of autophagy levels significantly attenuates disease-related insulin resistance in an AD rat model (Oh et al., 2023). Previous studies have suggested that autophagy disorders and defects may provide a possible explanation for the exacerbation of stress-induced insulin resistance in the islet cells of the endoplasmic reticulum (Zhou et al., 2009; Yang et al., 2010), whereas enhancing autophagy may effectively improve insulin sensitivity and cognitive defects (Wang et al., 2016). Exercise is considered the primary approach for activating autophagy, as it is a pivotal technique for consuming energy. Moreover, the role of exercise in disease prevention and treatment is inextricably associated with the regulation of autophagy (Sujkowski et al., 2022). In support of this concept, studies have demonstrated that exercise can initiate autophagy to improve insulin sensitivity by activating the interaction between AMPK and sestrins (Figure 3C) (Sujkowski et al., 2022). Researchers have also observed that exercise reduces the manifestation of insulin resistance by activating autophagy in the skeletal muscles of mice fed a high-fat diet (Cheng et al., 2022). Other studies have noted that exercise mitigates insulin resistance by stimulating the short-chain fatty acid (Yang et al., 2020) and Bcl-2 (He et al., 2012) pathways to activate autophagy. However, studies linking autophagy to insulin in neurodegenerative diseases remain limited. Nevertheless, regulation of autophagy in nervous system cells to improve insulin resistance may represent a potential strategy for managing neurodegenerative diseases.

### 6.4 Endoplasmic reticulum stress (ERS)

The endoplasmic reticulum (ER) is critical for protein synthesis, folding, and maturation. Nutrient deficiency, Ca^2+^ disorders, ROS, and glycosylation inhibition lead to increased endoplasmic reticulum misfolded proteins and trigger endoplasmic reticulum stress that in turn induces unfolded proteins, endoplasmic reticulum overload, and caspase-12-mediated responses to signal pathways such as the apoptotic pathway. Furthermore, damage to mitochondria-associated endoplasmic reticulum membranes caused by endoplasmic reticulum stress can promote the development of hypothalamic insulin resistance (Cheng et al., 2020). Experiments investigating the effect of inducing endoplasmic reticulum stress in POMC neurons observed inhibition of the activation of the insulin signaling pathway (Williams et al., 2014), whereas diabetes-related brain damage improved after inhibiting endoplasmic reticulum stress (Hu et al., 2020). Other researchers have revealed that the genetic deletion of the endoplasmic reticulum stress-related protein IRE1α and activating the PERK-mediated phosphorylation of FoXO can initiate and worsen the occurrence of insulin resistance (Zhang et al., 2013; Yao et al., 2017). Another possible mechanism of insulin resistance is the transfer of newly synthesized InsRs to the cell surface due to endoplasmic reticulum stress (Brown et al., 2020). A similar observation was noted in the hippocampal tissue (Sims-Robinson et al., 2016), where inhibiting endoplasmic reticulum stress reduced insulin resistance (Eo and Valentine, 2021). Furthermore, the activation of endoplasmic reticulum stress via the PERK-eIF2α pathway can result in insulin resistance (Li et al., 2015), whereas activating ATF6 can protect InsRs from the endoplasmic reticulum stress-mediated desensitization to a certain extent (Tang et al., 2011). Moreover, researchers have demonstrated that appropriate exercise can weaken the cellular response to endoplasmic reticulum stress (Marafon et al., 2022). Additional studies have suggested that exercise potentially reduces insulin resistance and cognitive impairment by improving endoplasmic reticulum stress (Figure 3D) (George et al., 2018). Exercise regulates endoplasmic reticulum stress, improves endoplasmic reticulum stress in skeletal muscles, and improves insulin resistance (Cheng et al., 2022). Similarly, endoplasmic reticulum stress detected in the liver and adipose tissue of obese rats improved with increased insulin sensitivity after exercise (da Luz et al., 2011). Therefore, exercise interventions to mitigate insulin resistance in neurodegenerative diseases may be intricately linked to a reduction in endoplasmic reticulum stress. Thus, the targeted amelioration of endoplasmic reticulum stress and insulin resistance requires further exploration in the field of neurodegeneration.

### 6.5 Reactive oxygen species (ROS)

ROS (including O₂•-, OH, and H₂O₂) are primarily generated during mitochondrial oxidative phosphorylation. Activation of the mitochondrial permeability transition pore and inner membrane anion channel (results in oxidation within and between the mitochondrial membranes. Moreover, ROS leakage can occur due to changes in the reduction environment (Zorov et al., 2014), wherein the mutual restriction of the oxidation and antioxidant systems essentially maintains normal intracellular ROS levels. Moderate concentrations of ROS are required for normal intracellular signaling. However, excessive ROS levels can induce inflammation, endoplasmic reticulum stress, and DNA damage and participate in molecular signaling pathways that damage the body’s environment and even trigger related diseases. Insulin resistance is a detrimental effect caused by altered ROS levels. ROS can inhibit the binding of IRS-1/2 to InsR by activating JNK, P38 MAPK, IKKβ, and PKCθ and inhibiting PTP1B and PTEN (Tiganis, 2011). Current research has demonstrated that exercise can induce a short-term increase in ROS levels; however, this alteration does not cause insulin resistance. However, exercise reduces ROS levels, due to the observation that exercise intensity is closely related, and reasonable long-term exercise may improve the antioxidant system of the body to manage oxidative stress (Figure 3E) (Nocella et al., 2019). Although few studies have been conducted examining the role of exercise in the contexts of regulating ROS levels, reducing insulin resistance, and alleviating neurodegenerative diseases, changes in ROS levels may represent a theoretically feasible mechanism to explain the applicability of exercise in the prevention and treatment of chronic neurodegenerative diseases.

## 7 Conclusion

Insulin resistance is an abnormal energy metabolic process that impairs the nutrient-absorbing ability of cells, ultimately resulting in abnormal physiological functions in the body. Insulin resistance is a crucial factor that promotes and exacerbates neurodegenerative diseases. In this review, we discuss insulin resistance in the context of prominent neurodegenerative diseases (Alzheimer’s disease, Parkinson’s disease, Huntington’s disease, Amyotrophic Lateral Sclerosis, and Multiple Sclerosis) and summarize the potential molecular mechanisms regulating exercise-associated insulin resistance in neurodegenerative diseases, and contribute to the improvement and alleviation of these disorders. Although exercise interventions may be challenging in some neurodegenerative diseases that progress rapidly, moderate exercise in the early stages of the disease may extend the treatment period and alleviate disease progression. Thus, improving insulin resistance via exercise may represent a valuable and essential intervention. Exercise may ameliorate neurodegenerative disorders by regulating mitochondrial quality, inflammation, autophagy, endoplasmic reticulum stress, and ROS production. However, studies investigating the relationship among neurodegenerative disorders, exercise, and insulin resistance are limited. Therefore, more in-depth exploratory research is required in this aspect, and this will help provide future directions for preventing and treating insulin resistance and neurodegenerative diseases via exercise.

## References

[B1] Abbasi F. Robakis T. K. Myoraku A. Watson K. T. Wroolie T. Rasgon N. L. (2023). Insulin resistance and accelerated cognitive aging. Psychoneuroendocrinology 147, 105944. 10.1016/j.psyneuen.2022.105944 36272362

[B2] Abbott M. A. Wells D. G. Fallon J. R. (1999). The insulin receptor tyrosine kinase substrate p58/53 and the insulin receptor are components of CNS synapses. J. Neurosci. 19, 7300–7308. 10.1523/JNEUROSCI.19-17-07300.1999 10460236 PMC6782521

[B3] Agrawal M. Saraf S. Saraf S. Antimisiaris S. G. Chougule M. B. Shoyele S. A. (2018). Nose-to-brain drug delivery: an update on clinical challenges and progress towards approval of anti-Alzheimer drugs. J. Control Release 281, 139–177. 10.1016/j.jconrel.2018.05.011 29772289

[B4] Andreassen O. A. Dedeoglu A. Stanojevic V. Hughes D. B. Browne S. E. Leech C. A. (2002). Huntington's disease of the endocrine pancreas: insulin deficiency and diabetes mellitus due to impaired insulin gene expression. Neurobiol. Dis. 11, 410–424. 10.1006/nbdi.2002.0562 12586550

[B5] Angulo J. El Assar M. Álvarez-Bustos A. Rodríguez-Mañas L. (2020). Physical activity and exercise: strategies to manage frailty. Redox Biol. 35, 101513. 10.1016/j.redox.2020.101513 32234291 PMC7284931

[B6] Arnold S. E. Arvanitakis Z. Macauley-Rambach S. L. Koenig A. M. Wang H. Y. Ahima R. S. (2018). Brain insulin resistance in type 2 diabetes and Alzheimer disease: concepts and conundrums. Nat. Rev. Neurol. 14, 168–181. 10.1038/nrneurol.2017.185 29377010 PMC6098968

[B7] Arora A. Dey C. S. (2016). SIRT2 regulates insulin sensitivity in insulin resistant neuronal cells. Biochem. Biophys. Res. Commun. 474, 747–752. 10.1016/j.bbrc.2016.05.029 27163642

[B8] Athauda D. Foltynie T. (2016). Insulin resistance and Parkinson's disease: a new target for disease modification? Prog. Neurobiol. 145-146, 98–120. 10.1016/j.pneurobio.2016.10.001 27713036

[B9] Ayer A. Fazakerley D. J. James D. E. Stocker R. (2022). The role of mitochondrial reactive oxygen species in insulin resistance. Free Radic. Biol. Med. 179, 339–362. 10.1016/j.freeradbiomed.2021.11.007 34775001

[B10] Ayromlou H. Hosseini S. Khalili M. Ayromlou S. Khamudchiyan S. Farajdokht F. (2023). Insulin resistance is associated with cognitive dysfunction in multiple sclerosis patients: a cross-sectional study. J. Neuroendocrinol. 35, e13288. 10.1111/jne.13288 37317829

[B11] Aziz N. A. Pijl H. Frölich M. Snel M. Streefland T. C. Roelfsema F. (2010). Systemic energy homeostasis in Huntington's disease patients. J. Neurol. Neurosurg. Psychiatry 81, 1233–1237. 10.1136/jnnp.2009.191833 20710011

[B12] Aziz N. A. Roos R. A. C. Pijl H. (2020). Insulin sensitivity in *de novo* Parkinson's disease: a hyperinsulinemic-euglycemic clamp study. Mov. Disord. 35, 1693–1694. 10.1002/mds.28181 32602947

[B13] Bacos K. Björkqvist M. Petersén A. Luts L. Maat-Schieman M. L. Roos R. A. (2008). Islet beta-cell area and hormone expression are unaltered in Huntington's disease. Histochem Cell Biol. 129, 623–629. 10.1007/s00418-008-0393-z 18259770

[B14] Baker L. D. Frank L. L. Foster-Schubert K. Green P. S. Wilkinson C. W. McTiernan A. (2010). Aerobic exercise improves cognition for older adults with glucose intolerance, a risk factor for Alzheimer's disease. J. Alzheimers Dis. 22, 569–579. 10.3233/JAD-2010-100768 20847403 PMC3049111

[B15] Banks W. A. Jaspan J. B. Huang W. Kastin A. J. (1997). Transport of insulin across the blood-brain barrier: saturability at euglycemic doses of insulin. Peptides 18, 1423–1429. 10.1016/s0196-9781(97)00231-3 9392846

[B16] Bassil F. Delamarre A. Canron M. H. Dutheil N. Vital A. Négrier-Leibreich M. L. (2022). Impaired brain insulin signalling in Parkinson's disease. Neuropathol. Appl. Neurobiol. 48, e12760. 10.1111/nan.12760 34405431

[B17] Bates G. P. Dorsey R. Gusella J. F. Hayden M. R. Kay C. Leavitt B. R. (2015). Huntington disease. Nat. Rev. Dis. Prim. 1, 15005. 10.1038/nrdp.2015.5 27188817

[B18] Baura G. D. Foster D. M. Porte D. Jr. Kahn S. E. Bergman R. N. Cobelli C. (1993). Saturable transport of insulin from plasma into the central nervous system of dogs *in vivo*. A mechanism for regulated insulin delivery to the brain. J. Clin. Invest. 92, 1824–1830. 10.1172/JCI116773 8408635 PMC288346

[B19] Benedict C. Brooks S. J. Kullberg J. Burgos J. Kempton M. J. Nordenskjöld R. (2012). Impaired insulin sensitivity as indexed by the HOMA score is associated with deficits in verbal fluency and temporal lobe gray matter volume in the elderly. Diabetes Care 35, 488–494. 10.2337/dc11-2075 22301128 PMC3322700

[B20] Benton C. R. Nickerson J. G. Lally J. Han X. X. Holloway G. P. Glatz J. F. (2008). Modest PGC-1alpha overexpression in muscle *in vivo* is sufficient to increase insulin sensitivity and palmitate oxidation in subsarcolemmal, not intermyofibrillar, mitochondria. J. Biol. Chem. 283, 4228–4240. 10.1074/jbc.M704332200 18079123

[B21] Benzler J. Ganjam G. K. Pretz D. Oelkrug R. Koch C. E. Legler K. (2015). Central inhibition of IKKβ/NF-κB signaling attenuates high-fat diet-induced obesity and glucose intolerance. Diabetes 64, 2015–2027. 10.2337/db14-0093 25626735

[B22] Björkqvist M. Petersén A. Bacos K. Isaacs J. Norlén P. Gil J. (2006). Progressive alterations in the hypothalamic-pituitary-adrenal axis in the R6/2 transgenic mouse model of Huntington's disease. Hum. Mol. Genet. 15, 1713–1721. 10.1093/hmg/ddl094 16613897

[B23] Bockmann J. Kreutz M. R. Gundelfinger E. D. Böckers T. M. (2002). ProSAP/Shank postsynaptic density proteins interact with insulin receptor tyrosine kinase substrate IRSp53. J. Neurochem. 83, 1013–1017. 10.1046/j.1471-4159.2002.01204.x 12421375

[B24] Boesgaard T. W. Nielsen T. T. Josefsen K. Hansen T. Jørgensen T. Pedersen O. (2009). Huntington's disease does not appear to increase the risk of diabetes mellitus. J. Neuroendocrinol. 21, 770–776. 10.1111/j.1365-2826.2009.01898.x 19602103

[B25] Bomfim T. R. Forny-Germano L. Sathler L. B. Brito-Moreira J. Houzel J. C. Decker H. (2012). An anti-diabetes agent protects the mouse brain from defective insulin signaling caused by Alzheimer's disease-associated Aβ oligomers. J. Clin. Invest. 122, 1339–1353. 10.1172/JCI57256 22476196 PMC3314445

[B26] Borai A. Livingstone C. Kaddam I. Ferns G. (2011). Selection of the appropriate method for the assessment of insulin resistance. BMC Med. Res. Methodol. 11, 158. 10.1186/1471-2288-11-158 22112229 PMC3258205

[B27] Bousquet M. St-Amour I. Vandal M. Julien P. Cicchetti F. Calon F. (2012). High-fat diet exacerbates MPTP-induced dopaminergic degeneration in mice. Neurobiol. Dis. 45, 529–538. 10.1016/j.nbd.2011.09.009 21971528

[B28] Braatz E. M. Coleman R. A. (2015). A mathematical model of insulin resistance in Parkinson's disease. Comput. Biol. Chem. 56, 84–97. 10.1016/j.compbiolchem.2015.04.003 25897824

[B29] Brown M. Dainty S. Strudwick N. Mihai A. D. Watson J. N. Dendooven R. (2020). Endoplasmic reticulum stress causes insulin resistance by inhibiting delivery of newly synthesized insulin receptors to the cell surface. Mol. Biol. Cell 31, 2597–2629. 10.1091/mbc.E18-01-0013 32877278 PMC7851869

[B30] Brüning J. C. Gautam D. Burks D. J. Gillette J. Schubert M. Orban P. C. (2000). Role of brain insulin receptor in control of body weight and reproduction. Science 289, 2122–2125. 10.1126/science.289.5487.2122 11000114

[B31] Cai J. Pires K. M. Ferhat M. Chaurasia B. Buffolo M. A. Smalling R. (2018). Autophagy ablation in adipocytes induces insulin resistance and reveals roles for lipid peroxide and Nrf2 signaling in adipose-liver crosstalk. Cell Rep. 25, 1708–1717. 10.1016/j.celrep.2018.10.040 30428342 PMC6802939

[B32] Cai Z. Xiao M. Chang L. Yan L. J. (2015). Role of insulin resistance in Alzheimer's disease. Metab. Brain Dis. 30, 839–851. 10.1007/s11011-014-9631-3 25399337

[B33] Chang C. C. Tsou S. H. Chen W. J. Ho Y. J. Hung H. C. Liu G. Y. (2021). miR-302 attenuates mutant huntingtin-induced cytotoxicity through restoration of autophagy and insulin sensitivity. Int. J. Mol. Sci. 22, 8424. 10.3390/ijms22168424 34445125 PMC8395150

[B34] Chaves G. Özel R. E. Rao N. V. Hadiprodjo H. Costa Y. D. Tokuno Z. (2017). Metabolic and transcriptomic analysis of Huntington's disease model reveal changes in intracellular glucose levels and related genes. Heliyon 3, e00381. 10.1016/j.heliyon.2017.e00381 28920088 PMC5576993

[B35] Chen Y. Zhao Y. Dai C. L. Liang Z. Run X. Iqbal K. (2014). Intranasal insulin restores insulin signaling, increases synaptic proteins, and reduces Aβ level and microglia activation in the brains of 3xTg-AD mice. Exp. Neurol. 261, 610–619. 10.1016/j.expneurol.2014.06.004 24918340

[B36] Cheng F. Dun Y. Cheng J. Ripley-Gonzalez J. W. Jiang W. You B. (2022). Exercise activates autophagy and regulates endoplasmic reticulum stress in muscle of high-fat diet mice to alleviate insulin resistance. Biochem. Biophys. Res. Commun. 601, 45–51. 10.1016/j.bbrc.2022.02.058 35228120

[B37] Cheng H. Gang X. He G. Liu Y. Wang Y. Zhao X. (2020). The molecular mechanisms underlying mitochondria-associated endoplasmic reticulum membrane-induced insulin resistance. Front. Endocrinol. (Lausanne) 11, 592129. 10.3389/fendo.2020.592129 33329397 PMC7719781

[B38] Chunchai T. Thunapong W. Yasom S. Wanchai K. Eaimworawuthikul S. Metzler G. (2018). Decreased microglial activation through gut-brain axis by prebiotics, probiotics, or synbiotics effectively restored cognitive function in obese-insulin resistant rats. J. Neuroinflammation 15, 11. 10.1186/s12974-018-1055-2 29316965 PMC5761137

[B39] Cieminski K. Flis D. J. Dzik K. Kaczor J. J. Czyrko E. Halon-Golabek M. (2021). Swim training affects Akt signaling and ameliorates loss of skeletal muscle mass in a mouse model of amyotrophic lateral sclerosis. Sci. Rep. 11, 20899. 10.1038/s41598-021-00319-1 34686697 PMC8536703

[B40] Cordeiro L. M. Machado M. L. da Silva A. F. Obetine Baptista F. B. da Silveira T. L. Soares F. A. A. (2020). Rutin protects huntington's disease through the insulin/IGF1 (IIS) signaling pathway and autophagy activity: study in caenorhabditis elegans model. Food Chem. Toxicol. 141, 111323. 10.1016/j.fct.2020.111323 32278002

[B41] Cui Y. Tang T. Y. Lu C. Q. Ju S. (2022). Insulin resistance and cognitive impairment: evidence from neuroimaging. J. Magn. Reson Imaging 56, 1621–1649. 10.1002/jmri.28358 35852470

[B42] Cuperfain A. B. Kennedy J. L. Gonçalves V. F. (2020). Overlapping mechanisms linking insulin resistance with cognition and neuroprogression in bipolar disorder. Neurosci. Biobehav Rev. 111, 125–134. 10.1016/j.neubiorev.2020.01.022 31978440

[B43] Dai C. Tan C. Zhao L. Liang Y. Liu G. Liu H. (2023). Glucose metabolism impairment in Parkinson's disease. Brain Res. Bull. 199, 110672. 10.1016/j.brainresbull.2023.110672 37210012

[B44] da Luz G. Frederico M. J. da Silva S. Vitto M. F. Cesconetto P. A. de Pinho R. A. (2011). Endurance exercise training ameliorates insulin resistance and reticulum stress in adipose and hepatic tissue in obese rats. Eur. J. Appl. Physiol. 111, 2015–2023. 10.1007/s00421-010-1802-2 21249392

[B45] Dela F. Helge J. W. (2013). Insulin resistance and mitochondrial function in skeletal muscle. Int. J. Biochem. Cell Biol. 45, 11–15. 10.1016/j.biocel.2012.09.019 23036788

[B46] de la Rubia Ortí J. E. Armero J. L. P. Sanchis-Sanchis C. E. Sancho-Castillo S. Salazar A. Caplliure-Llopis J. (2021). Muscle function differences between patients with bulbar and spinal onset amyotrophic lateral sclerosis. Does it depend on peripheral glucose? J. Clin. Med. 10, 1582. 10.3390/jcm10081582 33918552 PMC8069029

[B47] Denver P. English A. McClean P. L. (2018). Inflammation, insulin signaling and cognitive function in aged APP/PS1 mice. Brain Behav. Immun. 70, 423–434. 10.1016/j.bbi.2018.03.032 29604345

[B48] Doble B. W. Woodgett J. R. (2003). GSK-3: tricks of the trade for a multi-tasking kinase. J. Cell Sci. 116, 1175–1186. 10.1242/jcs.00384 12615961 PMC3006448

[B49] Dorsey E. R. Sherer T. Okun M. S. Bloem B. R. (2018). The emerging evidence of the Parkinson pandemic. J. Park. Dis. 8, S3–S8. 10.3233/JPD-181474 PMC6311367 30584159

[B50] Dou J. T. Chen M. Dufour F. Alkon D. L. Zhao W. Q. (2005). Insulin receptor signaling in long-term memory consolidation following spatial learning. Learn Mem. 12, 646–655. 10.1101/lm.88005 16287721 PMC1356184

[B51] Doust Y. V. Sumargo N. Ziebell J. M. Premilovac D. (2022). Insulin resistance in the brain: evidence supporting a role for inflammation, reactive microglia, and the impact of biological sex. Neuroendocrinology 112, 1027–1038. 10.1159/000524059 35279657

[B52] Emmanuel Y. Cochlin L. E. Tyler D. J. de Jager C. A. Smith A. D. Clarke K. (2013). Human hippocampal energy metabolism is impaired during cognitive activity in a lipid infusion model of insulin resistance. Brain Behav. 3, 134–144. 10.1002/brb3.124 23533158 PMC3607154

[B53] Eo H. Valentine R. J. (2021). Imoxin inhibits tunicamycin-induced endoplasmic reticulum stress and restores insulin signaling in C2C12 myotubes. Am. J. Physiol. Cell Physiol. 321, c221–c229. 10.1152/ajpcell.00544.2020 34077277

[B54] Evans J. L. Maddux B. A. Goldfine I. D. (2005). The molecular basis for oxidative stress-induced insulin resistance. Antioxid. Redox Signal 7, 1040–1052. 10.1089/ars.2005.7.1040 15998259

[B55] Fakih W. Zeitoun R. AlZaim I. Eid A. H. Kobeissy F. Abd-Elrahman K. S. (2022). Early metabolic impairment as a contributor to neurodegenerative disease: mechanisms and potential pharmacological intervention. Obes. (Silver Spring) 30, 982–993. 10.1002/oby.23400 35470973

[B56] Farokhi Larijani S. Hassanzadeh G. Zahmatkesh M. Radfar F. Farahmandfar M. (2023). Intranasal insulin intake and exercise improve memory function in amyloid-β induced Alzheimer's-like disease in rats: involvement of hippocampal BDNF-TrkB receptor. Behav. Brain Res. 460, 114814. 10.1016/j.bbr.2023.114814 38104636

[B57] Fernandez A. M. Torres-Alemán I. (2012). The many faces of insulin-like peptide signalling in the brain. Nat. Rev. Neurosci. 13, 225–239. 10.1038/nrn3209 22430016

[B58] Fiory F. Perruolo G. Cimmino I. Cabaro S. Pignalosa F. C. Miele C. (2019). The relevance of insulin action in the dopaminergic system. Front. Neurosci. 13, 868. 10.3389/fnins.2019.00868 31474827 PMC6706784

[B59] Frank S. Testa C. M. Stamler D. Kayson E. Davis C. Edmondson M. C. (2016). Effect of deutetrabenazine on chorea among patients with huntington disease: a randomized clinical trial. Jama 316, 40–50. 10.1001/jama.2016.8655 27380342

[B60] Frendo-Cumbo S. Jaldin-Fincati J. R. Coyaud E. Laurent E. M. N. Townsend L. K. Tan J. M. J. (2019). Deficiency of the autophagy gene ATG16L1 induces insulin resistance through KLHL9/KLHL13/CUL3-mediated IRS1 degradation. J. Biol. Chem. 294, 16172–16185. 10.1074/jbc.RA119.009110 31515271 PMC6827317

[B61] Gasparini L. Gouras G. K. Wang R. Gross R. S. Beal M. F. Greengard P. (2001). Stimulation of beta-amyloid precursor protein trafficking by insulin reduces intraneuronal beta-amyloid and requires mitogen-activated protein kinase signaling. J. Neurosci. 21, 2561–2570. 10.1523/JNEUROSCI.21-08-02561.2001 11306609 PMC6762523

[B62] Gasparini L. Netzer W. J. Greengard P. Xu H. (2002). Does insulin dysfunction play a role in Alzheimer's disease? Trends Pharmacol. Sci. 23, 288–293. 10.1016/s0165-6147(02)02037-0 12084635

[B63] George A. K. Behera J. Kelly K. E. Mondal N. K. Richardson K. P. Tyagi N. (2018). Exercise mitigates alcohol induced endoplasmic reticulum stress mediated cognitive impairment through ATF6-herp signaling. Sci. Rep. 8, 5158. 10.1038/s41598-018-23568-z 29581524 PMC5980102

[B64] Giannopapas V. Palaiodimou L. Kitsos D. Papagiannopoulou G. Stavrogianni K. Chasiotis A. (2023). The prevalence of diabetes mellitus type II (DMII) in the multiple sclerosis population: a systematic review and meta-analysis. J. Clin. Med. 12, 4948. 10.3390/jcm12154948 37568348 PMC10420178

[B65] Gustavsson A. Norton N. Fast T. Frölich L. Georges J. Holzapfel D. (2023). Global estimates on the number of persons across the Alzheimer's disease continuum. Alzheimers Dement. 19, 658–670. 10.1002/alz.12694 35652476

[B66] Haghikia A. Gold R. (2016). Positive Effect on multiple sclerosis with treatment of metabolic syndrome. JAMA Neurol. 73, 499–501. 10.1001/jamaneurol.2015.5050 26954938

[B67] Han D. H. Hancock C. R. Jung S. R. Higashida K. Kim S. H. Holloszy J. O. (2011). Deficiency of the mitochondrial electron transport chain in muscle does not cause insulin resistance. PLoS One 6, e19739. 10.1371/journal.pone.0019739 21589859 PMC3093385

[B68] Harno K. Rissanen A. Palo J. (1984). Glucose tolerance in amyotrophic lateral sclerosis. Acta Neurol. Scand. 70, 451–455. 10.1111/j.1600-0404.1984.tb00851.x 6516795

[B69] Harris M. D. Davidson M. B. Rosenberg C. S. (1986). Insulin antagonism is not a primary abnormality of amyotrophic lateral sclerois but is related to disease severity. J. Clin. Endocrinol. Metab. 63, 41–46. 10.1210/jcem-63-1-41 3519649

[B70] Havrankova J. Roth J. Brownstein M. (1978). Insulin receptors are widely distributed in the central nervous system of the rat. Nature 272, 827–829. 10.1038/272827a0 205798

[B71] He C. Bassik M. C. Moresi V. Sun K. Wei Y. Zou Z. (2012). Exercise-induced BCL2-regulated autophagy is required for muscle glucose homeostasis. Nature 481, 511–515. 10.1038/nature10758 22258505 PMC3518436

[B72] Hey-Mogensen M. Højlund K. Vind B. F. Wang L. Dela F. Beck-Nielsen H. (2010). Effect of physical training on mitochondrial respiration and reactive oxygen species release in skeletal muscle in patients with obesity and type 2 diabetes. Diabetologia 53, 1976–1985. 10.1007/s00125-010-1813-x 20526759

[B73] Hoeks J. Schrauwen P. (2012). Muscle mitochondria and insulin resistance: a human perspective. Trends Endocrinol. Metab. 23, 444–450. 10.1016/j.tem.2012.05.007 22726362

[B74] Holliday M. A. (1971). Metabolic rate and organ size during growth from infancy to maturity and during late gastation and early infancy. Pediatrics 47 (Suppl. 2), 169. 5551034

[B75] Holloszy J. O. (2013). Deficiency" of mitochondria in muscle does not cause insulin resistance. Diabetes 62, 1036–1040. 10.2337/db12-1107 23520283 PMC3609559

[B76] Hu T. Shi J. J. Fang J. Wang Q. Chen Y. B. Zhang S. J. (2020). Quercetin ameliorates diabetic encephalopathy through SIRT1/ER stress pathway in db/db mice. Aging (Albany NY) 12, 7015–7029. 10.18632/aging.103059 32312941 PMC7202537

[B77] Hurlbert M. S. Zhou W. Wasmeier C. Kaddis F. G. Hutton J. C. Freed C. R. (1999). Mice transgenic for an expanded CAG repeat in the Huntington's disease gene develop diabetes. Diabetes 48, 649–651. 10.2337/diabetes.48.3.649 10078572

[B78] Ispoglou T. Ferentinos P. Prokopidis K. Blake C. Aldrich L. Elia A. (2023). Exploring the impact of exercise and essential amino acid plus cholecalciferol supplementation on physical fitness and body composition in multiple sclerosis: a case study. Clin. Case Rep. 11, e7548. 10.1002/ccr3.7548 37323260 PMC10264925

[B79] Jannig P. R. Ruas J. L. (2017). Targeting mitochondrial mRNA translation to tackle obesity-induced insulin resistance: thumbs up for exercise. Acta Physiol. (Oxf) 219, 14–16. 10.1111/apha.12752 27419811

[B80] Jensen C. S. Musaeus C. S. Frikke-Schmidt R. Andersen B. B. Beyer N. Gottrup H. (2020). Physical exercise may increase plasma concentration of high-density lipoprotein-cholesterol in patients with Alzheimer's disease. Front. Neurosci. 14, 532. 10.3389/fnins.2020.00532 32536853 PMC7269030

[B81] Jia J. Wei C. Chen S. Li F. Tang Y. Qin W. (2018). The cost of Alzheimer's disease in China and re-estimation of costs worldwide. Alzheimers Dement. 14, 483–491. 10.1016/j.jalz.2017.12.006 29433981

[B82] Kahn B. B. (1996). Lilly lecture 1995. Glucose transport: pivotal step in insulin action. Diabetes 45, 1644–1654. 10.2337/diab.45.11.1644 8866574

[B83] Kakoty V. Kc S. Kumari S. Yang C. H. Dubey S. K. Sahebkar A. (2023a). Brain insulin resistance linked Alzheimer's and Parkinson's disease pathology: an undying implication of epigenetic and autophagy modulation. Inflammopharmacology 31, 699–716. 10.1007/s10787-023-01187-z 36952096

[B84] Kakoty V. Kc S. Yang C. H. Dubey S. K. Taliyan R. (2023b). Exploring the epigenetic regulated modulation of fibroblast growth factor 21 involvement in high-fat diet associated Parkinson's disease in rats. ACS Chem. Neurosci. 14, 725–740. 10.1021/acschemneuro.2c00659 36694924

[B85] Kang E. B. Cho J. Y. (2014). Effects of treadmill exercise on brain insulin signaling and β-amyloid in intracerebroventricular streptozotocin induced-memory impairment in rats. J. Exerc Nutr. Biochem. 18, 89–96. 10.5717/jenb.2014.18.1.89 PMC4241930 25566443

[B86] Kaspar B. K. Frost L. M. Christian L. Umapathi P. Gage F. H. (2005). Synergy of insulin-like growth factor-1 and exercise in amyotrophic lateral sclerosis. Ann. Neurol. 57, 649–655. 10.1002/ana.20451 15852403

[B87] Katsuki A. Yano Y. Sumida Y. Ito K. Fujii M. Tsuchihashi K. (1998). Significant decreased insulin secretion in a diabetic patient with clinically probable multiple sclerosis. Intern Med. 37, 865–869. 10.2169/internalmedicine.37.865 9840710

[B88] Kellar D. Craft S. (2020). Brain insulin resistance in Alzheimer's disease and related disorders: mechanisms and therapeutic approaches. Lancet Neurol. 19, 758–766. 10.1016/S1474-4422(20)30231-3 32730766 PMC9661919

[B89] Kelty T. J. Schachtman T. R. Mao X. Grigsby K. B. Childs T. E. Olver T. D. (2019). Resistance-exercise training ameliorates LPS-induced cognitive impairment concurrent with molecular signaling changes in the rat dentate gyrus. J. Appl. Physiol. 127, 254–263. 10.1152/japplphysiol.00249.2019 31120807

[B90] Khalili M. Khamodchyan S. Ayromlou H. Naser-Moghadasi A. (2020). Insulin resistance is related with cognition impairment in multiple sclerosis patients. Multiple Scler. Relat. Disord. 37, 101575. 10.1016/j.msard.2019.11.050

[B91] Kim S. H. Park S. S. Kim C. J. Kim T. W. (2022). Exercise with 40-Hz light flicker improves hippocampal insulin signaling in Alzheimer disease mice. J. Exerc Rehabil. 18, 20–27. 10.12965/jer.2244042.021 35356135 PMC8934612

[B92] Kleinridders A. Cai W. Cappellucci L. Ghazarian A. Collins W. R. Vienberg S. G. (2015). Insulin resistance in brain alters dopamine turnover and causes behavioral disorders. Proc. Natl. Acad. Sci. U. S. A. 112, 3463–3468. 10.1073/pnas.1500877112 25733901 PMC4371978

[B93] Kouchaki E. Tamtaji O. R. Salami M. Bahmani F. Daneshvar Kakhaki R. Akbari E. (2017). Clinical and metabolic response to probiotic supplementation in patients with multiple sclerosis: a randomized, double-blind, placebo-controlled trial. Clin. Nutr. 36, 1245–1249. 10.1016/j.clnu.2016.08.015 27669638

[B94] Krumpolec P. Vallova S. Slobodova L. Tirpakova V. Vajda M. Schon M. (2017). Aerobic-strength exercise improves metabolism and clinical state in Parkinson’s disease patients. Front. neurology 8, 698. 10.3389/fneur.2017.00698 PMC5743754 29312123

[B95] Kuga G. K. Muñoz V. R. Gaspar R. C. Nakandakari S. da Silva A. S. R. Botezelli J. D. (2018). Impaired insulin signaling and spatial learning in middle-aged rats: the role of PTP1B. Exp. Gerontol. 104, 66–71. 10.1016/j.exger.2018.02.005 29421605

[B96] Lalić N. M. Marić J. Svetel M. Jotić A. Stefanova E. Lalić K. (2008). Glucose homeostasis in Huntington disease: abnormalities in insulin sensitivity and early-phase insulin secretion. Archives Neurology 65, 476–480. 10.1001/archneur.65.4.476 18413469

[B97] Lee C. J. Sears C. L. Maruthur N. (2020). Gut microbiome and its role in obesity and insulin resistance. Ann. N. Y. Acad. Sci. 1461, 37–52. 10.1111/nyas.14107 31087391

[B98] Lee H. Ha T. Y. Jung C. H. Nirmala F. S. Park S. Y. Huh Y. H. (2021). Mitochondrial dysfunction in skeletal muscle contributes to the development of acute insulin resistance in mice. J. Cachexia Sarcopenia Muscle 12, 1925–1939. 10.1002/jcsm.12794 34605225 PMC8718067

[B99] Lee J. Pilch P. F. (1994). The insulin receptor: structure, function, and signaling. Am. J. Physiol. 266, C319–C334. 10.1152/ajpcell.1994.266.2.C319 8141246

[B100] Lei P. Ayton S. Finkelstein D. I. Spoerri L. Ciccotosto G. D. Wright D. K. (2012). Tau deficiency induces parkinsonism with dementia by impairing APP-mediated iron export. Nat. Med. 18, 291–295. 10.1038/nm.2613 22286308

[B101] LeWitt P. A. (2015). Levodopa therapy for Parkinson's disease: pharmacokinetics and pharmacodynamics. Mov. Disord. 30, 64–72. 10.1002/mds.26082 25449210

[B102] Li H. Zhou B. Liu J. Li F. Li Y. Kang X. (2015). Administration of progranulin (PGRN) triggers ER stress and impairs insulin sensitivity via PERK-eIF2α-dependent manner. Cell Cycle 14, 1893–1907. 10.1080/15384101.2015.1041686 26039714 PMC4615047

[B103] Li M. Chi X. Wang Y. Setrerrahmane S. Xie W. Xu H. (2022). Trends in insulin resistance: insights into mechanisms and therapeutic strategy. Signal Transduct. Target Ther. 7, 216. 10.1038/s41392-022-01073-0 35794109 PMC9259665

[B104] Li Y. Liu W. Li L. Hölscher C. (2016). Neuroprotective effects of a GIP analogue in the MPTP Parkinson's disease mouse model. Neuropharmacology 101, 255–263. 10.1016/j.neuropharm.2015.10.002 26453962

[B105] Liu Y. Zhou X. Xiao Y. Li C. Huang Y. Guo Q. (2020). miR-188 promotes liver steatosis and insulin resistance via the autophagy pathway. J. Endocrinol. 245, 411–423. 10.1530/JOE-20-0033 32252024

[B106] Luthi-Carter R. Taylor D. M. Pallos J. Lambert E. Amore A. Parker A. (2010). SIRT2 inhibition achieves neuroprotection by decreasing sterol biosynthesis. Proc. Natl. Acad. Sci. U. S. A. 107, 7927–7932. 10.1073/pnas.1002924107 20378838 PMC2867924

[B107] Lv H. Tang L. Guo C. Jiang Y. Gao C. Wang Y. (2020). Intranasal insulin administration may be highly effective in improving cognitive function in mice with cognitive dysfunction by reversing brain insulin resistance. Cogn. Neurodyn 14, 323–338. 10.1007/s11571-020-09571-z 32399074 PMC7203403

[B108] Ma T. C. Buescher J. L. Oatis B. Funk J. A. Nash A. J. Carrier R. L. (2007). Metformin therapy in a transgenic mouse model of Huntington's disease. Neurosci. Lett. 411, 98–103. 10.1016/j.neulet.2006.10.039 17110029

[B109] Mahalakshmi B. Maurya N. Lee S. D. Bharath Kumar V. (2020). Possible neuroprotective mechanisms of physical exercise in neurodegeneration. Int. J. Mol. Sci. 21, 5895. 10.3390/ijms21165895 32824367 PMC7460620

[B110] Malin S. K. Stewart N. R. Ude A. A. Alderman B. L. (2022). Brain insulin resistance and cognitive function: influence of exercise. J. Appl. Physiol. 133, 1368–1380. 10.1152/japplphysiol.00375.2022 36269295 PMC9744647

[B111] Marafon B. B. Pinto A. P. Ropelle E. R. de Moura L. P. Cintra D. E. Pauli J. R. (2022). Muscle endoplasmic reticulum stress in exercise. Acta Physiol. (Oxf) 235, e13799. 10.1111/apha.13799 35152547

[B112] Marciniak E. Leboucher A. Caron E. Ahmed T. Tailleux A. Dumont J. (2017). Tau deletion promotes brain insulin resistance. J. Exp. Med. 214, 2257–2269. 10.1084/jem.20161731 28652303 PMC5551570

[B113] Margolis R. U. Altszuler N. (1967). Insulin in the cerebrospinal fluid. Nature 215, 1375–1376. 10.1038/2151375a0 6055448

[B114] Medina A. Mahjoub Y. Shaver L. Pringsheim T. (2022). Prevalence and incidence of huntington's disease: an updated systematic review and meta-analysis. Mov. Disord. 37, 2327–2335. 10.1002/mds.29228 36161673 PMC10086981

[B115] Milanski M. Arruda A. P. Coope A. Ignacio-Souza L. M. Nunez C. E. Roman E. A. (2012). Inhibition of hypothalamic inflammation reverses diet-induced insulin resistance in the liver. Diabetes 61, 1455–1462. 10.2337/db11-0390 22522614 PMC3357298

[B116] Moosavi M. Naghdi N. Maghsoudi N. Zahedi Asl S. (2006). The effect of intrahippocampal insulin microinjection on spatial learning and memory. Horm. Behav. 50, 748–752. 10.1016/j.yhbeh.2006.06.025 16890939

[B117] Morris J. K. Bomhoff G. L. Stanford J. A. Geiger P. C. (2010). Neurodegeneration in an animal model of Parkinson's disease is exacerbated by a high-fat diet. Am. J. Physiol. Regul. Integr. Comp. Physiol. 299, R1082–R1090. 10.1152/ajpregu.00449.2010 20702796 PMC2957375

[B118] Morris J. K. Seim N. B. Bomhoff G. L. Geiger P. C. Stanford J. A. (2011). Effects of unilateral nigrostriatal dopamine depletion on peripheral glucose tolerance and insulin signaling in middle aged rats. Neurosci. Lett. 504, 219–222. 10.1016/j.neulet.2011.09.027 21964388 PMC3200476

[B119] Mowry E. Newsome S. Avornu A. Ravenna P. (2017). Intranasal insulin for improving cognitive function in multiple sclerosis. Multiple Scler. J. 18, e130681.

[B120] Nakatsuji H. Araki A. Hashizume A. Hijikata Y. Yamada S. Inagaki T. (2017). Correlation of insulin resistance and motor function in spinal and bulbar muscular atrophy. J. Neurol. 264, 839–847. 10.1007/s00415-017-8405-3 28229243

[B121] Nambron R. Silajdžić E. Kalliolia E. Ottolenghi C. Hindmarsh P. Hill N. R. (2016). A metabolic study of Huntington's disease. PLoS One 11, e0146480. 10.1371/journal.pone.0146480 26744893 PMC4706313

[B122] Nocella C. Cammisotto V. Pigozzi F. Borrione P. Fossati C. D'Amico A. (2019). Impairment between oxidant and antioxidant systems: short- and long-term implications for athletes' health. Nutrients 11, 1353. 10.3390/nu11061353 31208096 PMC6627820

[B123] Oh J. Park C. Kim S. Kim M. Kim C. S. Jo W. (2023). High levels of intracellular endotrophin in adipocytes mediate COPII vesicle supplies to autophagosome to impair autophagic flux and contribute to systemic insulin resistance in obesity. Metabolism 145, 155629. 10.1016/j.metabol.2023.155629 37302692

[B124] Özbeyli D. Sarı G. Özkan N. Karademir B. Yüksel M. Çilingir Kaya Ö T. (2017). Protective effects of different exercise modalities in an Alzheimer's disease-like model. Behav. Brain Res. 328, 159–177. 10.1016/j.bbr.2017.03.044 28390878

[B125] Pagel-Langenickel I. Bao J. Joseph J. J. Schwartz D. R. Mantell B. S. Xu X. (2008). PGC-1alpha integrates insulin signaling, mitochondrial regulation, and bioenergetic function in skeletal muscle. J. Biol. Chem. 283, 22464–22472. 10.1074/jbc.M800842200 18579525 PMC2504883

[B126] Park H. S. Cho H. S. Kim T. W. (2018). Physical exercise promotes memory capability by enhancing hippocampal mitochondrial functions and inhibiting apoptosis in obesity-induced insulin resistance by high fat diet. Metab. Brain Dis. 33, 283–292. 10.1007/s11011-017-0160-8 29185193

[B127] Park S. H. Nam G. E. Han K. Huh Y. Kim W. Lee M. K. (2021). Association of dynamic changes in metabolic syndrome status with the risk of Parkinson's disease: a nationwide cohort study. J. Park. Dis. 11, 1751–1759. 10.3233/JPD-212589 34120914

[B128] Patyn C. (2014) Impact of a high intensity training program on glucose tolerance in people with multiple sclerosis. Belgium: UHasselt.

[B129] Penesova A. Vlcek M. Imrich R. Vernerova L. Marko A. Meskova M. (2015). Hyperinsulinemia in newly diagnosed patients with multiple sclerosis. Metab. Brain Dis. 30, 895–901. 10.1007/s11011-015-9665-1 25809135

[B130] Peng Y. Liu J. Shi L. Tang Y. Gao D. Long J. (2016). Mitochondrial dysfunction precedes depression of AMPK/AKT signaling in insulin resistance induced by high glucose in primary cortical neurons. J. Neurochem. 137, 701–713. 10.1111/jnc.13563 26926143

[B131] Perruolo G. Viggiano D. Fiory F. Cassese A. Nigro C. Liotti A. (2016). Parkinson-like phenotype in insulin-resistant PED/PEA-15 transgenic mice. Sci. Rep. 6, 29967. 10.1038/srep29967 27426254 PMC4947959

[B132] Perurena O. H. Festoff B. W. (1987). Reduction in insulin receptors in amyotrophic lateral sclerosis correlates with reduced insulin sensitivity. Neurology 37, 1375–1379. 10.1212/wnl.37.8.1375 3614662

[B133] Pessin J. E. Thurmond D. C. Elmendorf J. S. Coker K. J. Okada S. (1999). Molecular basis of insulin-stimulated GLUT4 vesicle trafficking. Location! Location! Location. J. Biol. Chem. 274, 2593–2596. 10.1074/jbc.274.5.2593 9915783

[B134] Phillips O. R. Onopa A. K. Zaiko Y. V. Singh M. K. (2018). Insulin resistance is associated with smaller brain volumes in a preliminary study of depressed and obese children. Pediatr. Diabetes 19, 892–897. 10.1111/pedi.12672 29569318 PMC6030449

[B135] Poewe W. Seppi K. (2017). Insulin signalling: new target for Parkinson's treatments? Lancet 390, 1628–1630. 10.1016/S0140-6736(17)32101-3 28781109

[B136] Pradat P. F. Bruneteau G. Gordon P. H. Dupuis L. Bonnefont-Rousselot D. Simon D. (2010). Impaired glucose tolerance in patients with amyotrophic lateral sclerosis. Amyotroph. Lateral Scler. 11, 166–171. 10.3109/17482960902822960 20184518

[B137] Rabinowitz D. Zierler K. L. (1962). Forearm metabolism in obesity and its response to intra-arterial insulin. Characterization of insulin resistance and evidence for adaptive hyperinsulinism. J. Clin. Invest. 41, 2173–2181. 10.1172/JCI104676 13972875 PMC291152

[B138] Radikova Z. Penesova A. Vlcek M. Havranova A. Sivakova M. Siarnik P. (2018). LDL and HDL lipoprotein subfractions in multiple sclerosis patients with decreased insulin sensitivity. Endocr. Regul. 52, 139–145. 10.2478/enr-2018-0017 31517604

[B139] Rahmati M. Keshvari M. Mirnasouri R. Chehelcheraghi F. (2021). Exercise and Urtica dioica extract ameliorate hippocampal insulin signaling, oxidative stress, neuroinflammation, and cognitive function in STZ-induced diabetic rats. Biomed. Pharmacother. 139, 111577. 10.1016/j.biopha.2021.111577 33839493

[B140] Reger M. A. Watson G. S. Green P. S. Baker L. D. Cholerton B. Fishel M. A. (2008a). Intranasal insulin administration dose-dependently modulates verbal memory and plasma amyloid-beta in memory-impaired older adults. J. Alzheimers Dis. 13, 323–331. 10.3233/jad-2008-13309 18430999 PMC2804944

[B141] Reger M. A. Watson G. S. Green P. S. Wilkinson C. W. Baker L. D. Cholerton B. (2008b). Intranasal insulin improves cognition and modulates beta-amyloid in early AD. Neurology 70, 440–448. 10.1212/01.WNL.0000265401.62434.36 17942819

[B142] Reyes E. T. Perurena O. H. Festoff B. W. Jorgensen R. Moore W. V. (1984). Insulin resistance in amyotrophic lateral sclerosis. J. Neurol. Sci. 63, 317–324. 10.1016/0022-510x(84)90154-0 6374040

[B143] Roberts C. K. Hevener A. L. Barnard R. J. (2013). Metabolic syndrome and insulin resistance: underlying causes and modification by exercise training. Compr. Physiol. 3, 1–58. 10.1002/cphy.c110062 23720280 PMC4129661

[B144] Rodriguez-Araujo G. Nakagami H. Takami Y. Katsuya T. Akasaka H. Saitoh S. (2015). Low alpha-synuclein levels in the blood are associated with insulin resistance. Sci. Rep. 5, 12081. 10.1038/srep12081 26159928 PMC4498217

[B145] Rorato R. Borges B. C. Uchoa E. T. Antunes-Rodrigues J. Elias C. F. Elias L. L. K. (2017). LPs-induced low-grade inflammation increases hypothalamic JNK expression and causes central insulin resistance irrespective of body weight changes. Int. J. Mol. Sci. 18, 1431. 10.3390/ijms18071431 28677618 PMC5535922

[B146] Rothstein J. D. (2017). Edaravone: a new drug approved for ALS. Cell 171, 725. 10.1016/j.cell.2017.10.011 29100067

[B147] Rudnicka E. Napierała P. Podfigurna A. Męczekalski B. Smolarczyk R. Grymowicz M. (2020). The World Health Organization (WHO) approach to healthy ageing. Maturitas 139, 6–11. 10.1016/j.maturitas.2020.05.018 32747042 PMC7250103

[B148] Ruegsegger G. N. Creo A. L. Cortes T. M. Dasari S. Nair K. S. (2018). Altered mitochondrial function in insulin-deficient and insulin-resistant states. J. Clin. Invest. 128, 3671–3681. 10.1172/JCI120843 30168804 PMC6118582

[B149] Ruegsegger G. N. Vanderboom P. M. Dasari S. Klaus K. A. Kabiraj P. McCarthy C. B. (2019). Exercise and metformin counteract altered mitochondrial function in the insulin-resistant brain. JCI Insight 4, e130681. 10.1172/jci.insight.130681 31534057 PMC6795285

[B150] Ruiz-Argüelles A. Méndez-Huerta M. A. Lozano C. D. Ruiz-Argüelles G. J. (2018). Metabolomic profile of insulin resistance in patients with multiple sclerosis is associated to the severity of the disease. Mult. Scler. Relat. Disord. 25, 316–321. 10.1016/j.msard.2018.08.014 30193201

[B151] Russo C. V. Salvatore E. Saccà F. Tucci T. Rinaldi C. Sorrentino P. (2013). Insulin sensitivity and early-phase insulin secretion in normoglycemic Huntington's disease patients. J. Huntingt. Dis. 2, 501–507. 10.3233/JHD-130078 25062734

[B152] Sadegh S. Golestany A. (2017). Effects of 10 weeks of aerobic training in water on chemerin and insulin resistance in women with multiple sclerosis. Intern. Med. Today 23, 226–234.

[B153] Sánchez-Gómez A. Alcarraz-Vizán G. Fernández M. Fernández-Santiago R. Ezquerra M. Cámara A. (2020). Peripheral insulin and amylin levels in Parkinson's disease. Park. Relat. Disord. 79, 91–96. 10.1016/j.parkreldis.2020.08.018 32911247

[B154] Sangwung P. Petersen K. F. Shulman G. I. Knowles J. W. (2020). Mitochondrial dysfunction, insulin resistance, and potential genetic implications. Endocrinology 161, bqaa017. 10.1210/endocr/bqaa017 32060542 PMC7341556

[B155] Schiöth H. B. Frey W. H. Brooks S. J. Benedict C. (2012). Insulin to treat Alzheimer's disease: just follow your nose? Expert Rev. Clin. Pharmacol. 5, 17–20. 10.1586/ecp.11.70 22142155

[B156] Schubert M. Brazil D. P. Burks D. J. Kushner J. A. Ye J. Flint C. L. (2003). Insulin receptor substrate-2 deficiency impairs brain growth and promotes tau phosphorylation. J. Neurosci. 23, 7084–7092. 10.1523/JNEUROSCI.23-18-07084.2003 12904469 PMC6740672

[B157] Schubert M. Gautam D. Surjo D. Ueki K. Baudler S. Schubert D. (2004). Role for neuronal insulin resistance in neurodegenerative diseases. Proc. Natl. Acad. Sci. U. S. A. 101, 3100–3105. 10.1073/pnas.0308724101 14981233 PMC365750

[B158] Sharma S. Taliyan R. (2018). High fat diet feeding induced insulin resistance exacerbates 6-OHDA mediated neurotoxicity and behavioral abnormalities in rats. Behav. Brain Res. 351, 17–23. 10.1016/j.bbr.2018.05.025 29842916

[B159] Shi H. Kokoeva M. V. Inouye K. Tzameli I. Yin H. Flier J. S. (2006). TLR4 links innate immunity and fatty acid-induced insulin resistance. J. Clin. Invest. 116, 3015–3025. 10.1172/JCI28898 17053832 PMC1616196

[B160] Shimizu T. Honda M. Ohashi T. Tsujino M. Nagaoka U. Kawata A. (2011). Hyperosmolar hyperglycemic state in advanced amyotrophic lateral sclerosis. Amyotroph. Lateral Scler. 12, 379–381. 10.3109/17482968.2010.539234 21126160

[B161] Shoelson S. E. Lee J. Goldfine A. B. (2006). Inflammation and insulin resistance. J. Clin. Invest. 116, 1793–1801. 10.1172/JCI29069 16823477 PMC1483173

[B162] Shonesy B. C. Thiruchelvam K. Parameshwaran K. Rahman E. A. Karuppagounder S. S. Huggins K. W. (2012). Central insulin resistance and synaptic dysfunction in intracerebroventricular-streptozotocin injected rodents. Neurobiol. Aging 33, 430.e435–18. 10.1016/j.neurobiolaging.2010.12.002 21256630

[B163] Siddiqui N. Ali J. Parvez S. Zameer S. Najmi A. K. Akhtar M. (2021). Linagliptin, a DPP-4 inhibitor, ameliorates Aβ (1-42) peptides induced neurodegeneration and brain insulin resistance (BIR) via insulin receptor substrate-1 (IRS-1) in rat model of Alzheimer's disease. Neuropharmacology 195, 108662. 10.1016/j.neuropharm.2021.108662 34119519

[B164] Sims-Robinson C. Bakeman A. Glasser R. Boggs J. Pacut C. Feldman E. L. (2016). The role of endoplasmic reticulum stress in hippocampal insulin resistance. Exp. Neurol. 277, 261–267. 10.1016/j.expneurol.2016.01.007 26775176 PMC4802497

[B165] Soke F. Kocer B. Fidan I. Keskinoglu P. Guclu-Gunduz A. (2021). Effects of task-oriented training combined with aerobic training on serum BDNF, GDNF, IGF-1, VEGF, TNF-α, and IL-1β levels in people with Parkinson's disease: a randomized controlled study. Exp. Gerontol. 150, 111384. 10.1016/j.exger.2021.111384 33965556

[B166] Soliman R. H. Farhan H. M. Hegazy M. Oraby M. I. Kamel S. H. Hassan A. (2020). Impact of insulin resistance and metabolic syndrome on disability in patients with multiple sclerosis. Egypt. J. Neurology, Psychiatry Neurosurg. 56, 18. 10.1186/s41983-020-0155-y

[B167] Song J. Kang S. M. Kim E. Kim C. H. Song H. T. Lee J. E. (2015). Impairment of insulin receptor substrate 1 signaling by insulin resistance inhibits neurite outgrowth and aggravates neuronal cell death. Neuroscience 301, 26–38. 10.1016/j.neuroscience.2015.05.072 26047734

[B168] Speziale R. Montesano C. Di Pietro G. Cicero D. O. Summa V. Monteagudo E. (2023). The urine metabolome of R6/2 and zQ175DN Huntington's disease mouse models. Metabolites 13, 961. 10.3390/metabo13080961 37623904 PMC10456449

[B169] Spielman L. J. Little J. P. Klegeris A. (2014). Inflammation and insulin/IGF-1 resistance as the possible link between obesity and neurodegeneration. J. Neuroimmunol. 273, 8–21. 10.1016/j.jneuroim.2014.06.004 24969117

[B170] Stenvers D. J. Scheer F. Schrauwen P. la Fleur S. E. Kalsbeek A. (2019). Circadian clocks and insulin resistance. Nat. Rev. Endocrinol. 15, 75–89. 10.1038/s41574-018-0122-1 30531917

[B171] Stuckenschneider T. Abeln V. Foitschik T. Abel T. Polidori M. C. Strüder H. K. (2021). Disease-inclusive exercise classes improve physical fitness and reduce depressive symptoms in individuals with and without Parkinson's disease-A feasibility study. Brain Behav. 11, e2352. 10.1002/brb3.2352 34472722 PMC8553328

[B172] Sujkowski A. Hong L. Wessells R. J. Todi S. V. (2022). The protective role of exercise against age-related neurodegeneration. Ageing Res. Rev. 74, 101543. 10.1016/j.arr.2021.101543 34923167 PMC8761166

[B173] Szymura J. Kubica J. Wiecek M. Pera J. (2020). The immunomodulary effects of systematic exercise in older adults and people with Parkinson's disease. J. Clin. Med. 9, 184. 10.3390/jcm9010184 31936624 PMC7019419

[B174] Talbot K. Wang H. Y. Kazi H. Han L. Y. Bakshi K. P. Stucky A. (2012). Demonstrated brain insulin resistance in Alzheimer's disease patients is associated with IGF-1 resistance, IRS-1 dysregulation, and cognitive decline. J. Clin. Invest. 122, 1316–1338. 10.1172/JCI59903 22476197 PMC3314463

[B175] Tang X. Shen H. Chen J. Wang X. Zhang Y. Chen L. L. (2011). Activating transcription factor 6 protects insulin receptor from ER stress-stimulated desensitization via p42/44 ERK pathway. Acta Pharmacol. Sin. 32, 1138–1147. 10.1038/aps.2011.75 21841811 PMC4003301

[B176] The Lancet N. (2021). Multiple sclerosis under the spotlight. Lancet Neurol. 20, 497. 10.1016/S1474-4422(21)00170-8 34146496

[B177] Tiganis T. (2011). Reactive oxygen species and insulin resistance: the good, the bad and the ugly. Trends Pharmacol. Sci. 32, 82–89. 10.1016/j.tips.2010.11.006 21159388

[B178] Tsai C. P. Hu C. Lee C. T. (2019). Finding diseases associated with amyotrophic lateral sclerosis: a total population-based case-control study. Amyotroph. Lateral Scler. Front. Degener. 20, 82–89. 10.1080/21678421.2018.1522354 30422689

[B179] Um H. S. Kang E. B. Leem Y. H. Cho I. H. Yang C. H. Chae K. R. (2008). Exercise training acts as a therapeutic strategy for reduction of the pathogenic phenotypes for Alzheimer's disease in an NSE/APPsw-transgenic model. Int. J. Mol. Med. 22, 529–539. 18813861

[B180] United Nations Population Division (2002) Aging of the world’s population, 1950-2050. United Nations Publications, 1–483.

[B181] Waldron A. M. Wintmolders C. Bottelbergs A. Kelley J. B. Schmidt M. E. Stroobants S. (2015). *In vivo* molecular neuroimaging of glucose utilization and its association with fibrillar amyloid-β load in aged APPPS1-21 mice. Alzheimers Res. Ther. 7, 76. 10.1186/s13195-015-0158-6 26666747 PMC4678474

[B182] Wang M. Zhang H. Liang J. Huang J. Chen N. (2023). Exercise suppresses neuroinflammation for alleviating Alzheimer's disease. J. Neuroinflammation 20, 76. 10.1186/s12974-023-02753-6 36935511 PMC10026496

[B183] Wang Y. Hu Y. Sun C. Zhuo S. He Z. Wang H. (2016). Down-regulation of Risa improves insulin sensitivity by enhancing autophagy. Faseb J. 30, 3133–3145. 10.1096/fj.201500058R 27251173

[B184] Watson G. S. Peskind E. R. Asthana S. Purganan K. Wait C. Chapman D. (2003). Insulin increases CSF Abeta42 levels in normal older adults. Neurology 60, 1899–1903. 10.1212/01.wnl.0000065916.25128.25 12821730

[B185] Wens I. Dalgas U. Vandenabeele F. Verboven K. Hansen D. Deckx N. (2017). High intensity aerobic and resistance exercise can improve glucose tolerance in persons with multiple sclerosis: a randomized controlled trial. Am. J. Phys. Med. Rehabil. 96, 161–166. 10.1097/PHM.0000000000000563 27362697

[B186] Wens I. Hansen D. Verboven K. Deckx N. Kosten L. Stevens A. L. (2015). Impact of 24 weeks of resistance and endurance exercise on glucose tolerance in persons with multiple sclerosis. Am. J. Phys. Med. Rehabil. 94, 838–847. 10.1097/PHM.0000000000000257 25768064

[B187] Werther G. A. Hogg A. Oldfield B. J. McKinley M. J. Figdor R. Mendelsohn F. A. (1989). Localization and characterization of insulin-like growth factor-I receptors in rat brain and pituitary gland using *in vitro* autoradiography and computerized densitometry* A distinct distribution from insulin receptors. J. Neuroendocrinol. 1, 369–377. 10.1111/j.1365-2826.1989.tb00131.x 19210430

[B188] Wijtenburg S. A. Kapogiannis D. Korenic S. A. Mullins R. J. Tran J. Gaston F. E. (2019). Brain insulin resistance and altered brain glucose are related to memory impairments in schizophrenia. Schizophr. Res. 208, 324–330. 10.1016/j.schres.2019.01.031 30760413 PMC6656556

[B189] Willette A. A. Johnson S. C. Birdsill A. C. Sager M. A. Christian B. Baker L. D. (2015). Insulin resistance predicts brain amyloid deposition in late middle-aged adults. Alzheimers Dement. 11, 504–510. 10.1016/j.jalz.2014.03.011 25043908 PMC4297592

[B190] Williams K. W. Liu T. Kong X. Fukuda M. Deng Y. Berglund E. D. (2014). Xbp1s in Pomc neurons connects ER stress with energy balance and glucose homeostasis. Cell Metab. 20, 471–482. 10.1016/j.cmet.2014.06.002 25017942 PMC4186248

[B191] Woods S. C. Seeley R. J. Baskin D. G. Schwartz M. W. (2003). Insulin and the blood-brain barrier. Curr. Pharm. Des. 9, 795–800. 10.2174/1381612033455323 12678878

[B192] Wu H. Wang Y. Li W. Chen H. Du L. Liu D. (2019). Deficiency of mitophagy receptor FUNDC1 impairs mitochondrial quality and aggravates dietary-induced obesity and metabolic syndrome. Autophagy 15, 1882–1898. 10.1080/15548627.2019.1596482 30898010 PMC6844496

[B193] Xie L. Helmerhorst E. Taddei K. Plewright B. Van Bronswijk W. Martins R. (2002). Alzheimer's beta-amyloid peptides compete for insulin binding to the insulin receptor. J. Neurosci. 22, Rc221. 10.1523/JNEUROSCI.22-10-j0001.2002 12006603 PMC6757630

[B194] Xin W. Li Z. Xu Y. Yu Y. Zhou Q. Chen L. (2016). Autophagy protects human podocytes from high glucose-induced injury by preventing insulin resistance. Metabolism 65, 1307–1315. 10.1016/j.metabol.2016.05.015 27506738

[B195] Xu L. Liu T. Liu L. Yao X. Chen L. Fan D. (2020). Global variation in prevalence and incidence of amyotrophic lateral sclerosis: a systematic review and meta-analysis. J. Neurol. 267, 944–953. 10.1007/s00415-019-09652-y 31797084

[B196] Yang L. Li P. Fu S. Calay E. S. Hotamisligil G. S. (2010). Defective hepatic autophagy in obesity promotes ER stress and causes insulin resistance. Cell Metab. 11, 467–478. 10.1016/j.cmet.2010.04.005 20519119 PMC2881480

[B197] Yang L. Zhang X. Li S. Wang H. Zhang X. Liu L. (2020). Intranasal insulin ameliorates cognitive impairment in a rat model of Parkinson's disease through Akt/GSK3β signaling pathway. Life Sci. 259, 118159. 10.1016/j.lfs.2020.118159 32763288

[B198] Yano H. Seino Y. Inagaki N. Hinokio Y. Yamamoto T. Yasuda K. (1991). Tissue distribution and species difference of the brain type glucose transporter (GLUT3). Biochem. Biophys. Res. Commun. 174, 470–477. 10.1016/0006-291x(91)91440-n 1704223

[B199] Yao T. Deng Z. Gao Y. Sun J. Kong X. Huang Y. (2017). Ire1α in pomc neurons is required for thermogenesis and glycemia. Diabetes 66, 663–673. 10.2337/db16-0533 28028078 PMC5319716

[B200] Yoon S. O. Park D. J. Ryu J. C. Ozer H. G. Tep C. Shin Y. J. (2012). JNK3 perpetuates metabolic stress induced by Aβ peptides. Neuron 75, 824–837. 10.1016/j.neuron.2012.06.024 22958823 PMC3438522

[B201] Zabielski P. Lanza I. R. Gopala S. Heppelmann C. J. Bergen H. R. Dasari S. (2016). Altered skeletal muscle mitochondrial proteome as the basis of disruption of mitochondrial function in diabetic mice. Diabetes 65, 561–573. 10.2337/db15-0823 26718503 PMC4764144

[B202] Zhang H. Liang J. L. Wu Q. Y. Li J. X. Liu Y. Wu L. W. (2022b). Swimming suppresses cognitive decline of HFD-induced obese mice through reversing hippocampal inflammation, insulin resistance, and BDNF level. Nutrients 14, 2432. 10.3390/nu14122432 35745162 PMC9228449

[B203] Zhang W. Hietakangas V. Wee S. Lim S. C. Gunaratne J. Cohen S. M. (2013). ER stress potentiates insulin resistance through PERK-mediated FOXO phosphorylation. Genes Dev. 27, 441–449. 10.1101/gad.201731.112 23431056 PMC3589560

[B204] Zhang Y. Wang R. Fu X. Song H. (2022a). Non-insulin-based insulin resistance indexes in predicting severity for coronary artery disease. Diabetol. Metab. Syndr. 14, 191. 10.1186/s13098-022-00967-x 36528713 PMC9759860

[B205] Zhao W. Q. Chen H. Quon M. J. Alkon D. L. (2004). Insulin and the insulin receptor in experimental models of learning and memory. Eur. J. Pharmacol. 490, 71–81. 10.1016/j.ejphar.2004.02.045 15094074

[B206] Zhou L. Zhang J. Fang Q. Liu M. Liu X. Jia W. (2009). Autophagy-mediated insulin receptor down-regulation contributes to endoplasmic reticulum stress-induced insulin resistance. Mol. Pharmacol. 76, 596–603. 10.1124/mol.109.057067 19541767 PMC2730390

[B207] Zorov D. B. Juhaszova M. Sollott S. J. (2014). Mitochondrial reactive oxygen species (ROS) and ROS-induced ROS release. Physiol. Rev. 94, 909–950. 10.1152/physrev.00026.2013 24987008 PMC4101632

